# Supplementary material for: Infection control in the home: a qualitative study exploring perceptions and experiences of adhering to protective behaviours in the home during the COVID-19 pandemic
Source: BMJ Open. 2021 Dec 1;11(12):e056161. doi: 10.1136/bmjopen-2021-056161 (PMC8637310; doi:10.1136/bmjopen-2021-056161)
Supplement: Supplementary data [file bmjopen-2021-056161supp001.pdf]

Supplementary File 1

Coding Manual

**Theme**	**Subthemes**	**Definition**	**Example quote**
**Perceived risk**	Current levels of virus in circulation	Weighing up perceived risk of virus based on current infection rates	“*I want to know is it safe for me to go out? You know, what’s the transmission rate where I live?”* (int 6)
	Perceived likelihood of virus entering the home	Likelihood of a household member or someone outside the home bringing the virus into their home	*“the biggest concern is just bringing it in from outside, but I’m not doing particularly too much to risk that at the moment, I wouldn’t say”.* (int 9)
	Perceived risk of severe consequences to health	Considering how vulnerable household members are to becoming severely ill from the virus	*“I’m fifty-three, going on fifty-four, and the age group is looking not so brilliant now, when I was looking in press reports. I know they said over seventy. I’m pretty fit, which is good. The only problem is, I have high blood pressure, and I’m on medication for that”.* (int 5)
**Belief in the effectiveness of the protective behaviours**	Perceived value of cleaning	Perceived need and motivation to clean surfaces and hands based on perceived effectiveness	*“I mean, I always washed my hands a lot, obviously, working in a tea room, so I had to wash my hands all the time anyway. So I obviously now wash them for longer, and more carefully. And I also am very, very, very conscious now of whether I’m touching my face or… things like that.*” (int 3)
	Perceived value of face-coverings	Perceived need and motivation to wear	*“The thing with the face coverings is, they haven’t got any filters in them, these cloth ones…. And I think it*

		face-covering based on perceive effectiveness	*could be more infectious, because it'll get wet with your breathing. And then it's no good to anyone"*. (int 6)
	Barrier: Virus is likely to spread before you know you're ill	Belief in the potential to contain the virus if it enters the home	*"do everything in my power to prevent getting it, by assuming that I haven't got it, so wearing a mask in the house, keeping two metres, trying to keep the person that's got it in the household in a separate room"* (int 9)
	Facilitator: Reducing all or nothing thinking	Perception that it is worth reducing exposure to minimise viral load, and that even small changes can make a difference	*"the less we get in touch- the less we have contact with the virus, the safer we will be"* (int 7)
**Acceptability of distancing and isolation**	Barrier: Importance of time together	Includes concerns about own or others' mental well-being if spending time apart, and the value placed on time spent together.	*"I'm worried more about like the mental health of the other people. So although we're very careful, and not mixing. So I don't think I could cut down on the amount of time I spend with other people, because they'll get lonely"* (int 5)<br><br>*"Staying 2m from my 11yr old means me pushing him away and not sharing our only front room seating with him."* (s18)
	Facilitator: Ways of maintaining (distanced) intimacy	Finding ways to maintain emotional intimacy when social distancing or self-isolating	*"We have, I had a bit of, as I said, a bit of a dry cuddle, like I go over his shoulders, but I don't breathe on him and he doesn't breathe on me. So*

			we're kind of on board with it, you know?" (int 2)
**Having capacity to perform protective behaviours**		Factors which reduce a person's capacity to engage with and perform the behaviours. For example; having enough space, or not, in your house to either socially distance or self-isolate. Includes descriptions of encouraging others to adhere to protective behaviours, or the challenges of trying to influence others.	*"My son sits in one settee and my husband and I sit in the other. And that… it doesn't protect us all, we're not all sitting on our own sofa, but who has three sofas in their room? So… we do what we can".* (int 7)<br>*"I will just keep reminding him, all the time, to wash his hands. And he'll say, "I've done it." You say, "No you haven't. The sink's not wet." And, "well I did it. I did do it, I did it when I got to my...' Because he's a sink in his room, "I did it when I got to my room" which we know is not necessarily the case".* (int 1)<br>*"I tried to implement the isolation of amazon parcels and mocked by my teenage daughter!"* (s46)
**Habit forming reduces effort**		Descriptions of behaviours becoming easier when highly practiced/ingrained	*"Anything I can wipe down, I wipe down. So that, now… it, I mean, it is… it still is harder than it used to be, because I never would've done that before. But it is more normal now".* (int 3)<br>*"Good advice needs to be followed but sometimes we forget. It needs to be made into a habit."* (s78)
**Confidence in how to perform the behaviours**		Inconsistent and confusing information undermines confidence. Concise information	"*I've just read an article in the Times that this [washing fresh produce in soapy water] is very dangerous, so I'll have to re-read it and decide, or*

		and validation increases confidence in how to perform the behaviours	*maybe you can tell me, because I've got no idea now, I'm completely confused"*. (int 5)<br><br>"*So it's more if that, that's… it's attractiveness of thinking, 'yes, I… you know, if that's the kind of thing I'm doing and, you know, health experts are saying yeah, that's right...*" (int 2)<br><br>*"The advice helps to empower you, that we are not completely defenseless against deadly germs, we can be pro- active in stopping these germs making us ill.."* (s110)
**Social norms affect motivation to engage in the behaviours**		Includes any descriptions about others' perceived behaviour and its' effect on people's willingness to perform effortful behaviours	*"It starts being more relatable, and you can start imagining yourself putting those changes into practice. Like when the guy said his wife is always going round with the anti-bac, you can sort of imagine yourself doing that."* (int 12)
