# Supplementary material for: Infection control in the home: a qualitative study exploring perceptions and experiences of adhering to protective behaviours in the home during the COVID-19 pandemic
Source: BMJ Open. 2021 Dec 1;11(12):e056161. doi: 10.1136/bmjopen-2021-056161 (PMC8637310; doi:10.1136/bmjopen-2021-056161)
Supplement: Author's manuscript [file bmjopen-2021-056161.draft_revisions.pdf]

BMJ Open

BMJ Open is committed to open peer review. As part of this commitment we make the peer review history of every article we publish publicly available.

When an article is published we post the peer reviewers' comments and the authors' responses online. We also post the versions of the paper that were used during peer review. These are the versions that the peer review comments apply to.

The versions of the paper that follow are the versions that were submitted during the peer review process. They are not the versions of record or the final published versions. They should not be cited or distributed as the published version of this manuscript.

BMJ Open is an open access journal and the full, final, typeset and author-corrected version of record of the manuscript is available on our site with no access controls, subscription charges or pay-per-view fees (http://bmjopen.bmj.com).

If you have any questions on BMJ Open's open peer review process please email info.bmjopen@bmj.com

Infection control in the home: A qualitative study exploring perceptions and experiences of adhering to protective behaviours in the home during the COVID-19 pandemic

Journal:	*BMJ Open*
Manuscript ID	bmjopen-2021-056161
Article Type:	Original research
Date Submitted by the Author:	04-Aug-2021
Complete List of Authors:	Morton, Katherine; University of Southampton, School of Psychology<br>Towler, Lauren; University of Southampton, School of Psychology<br>Groot, Julia; University of Bath, Department of Psychology<br>Miller, Sascha; University of Southampton, School of Psychology<br>Ainsworth, Ben; University of Bath, Department of Psychology; University of Southampton, NIHR Biomedical Research Centre, Faculty of Medicine<br>Denison-Day, James; University of Southampton, School of Psychology<br>Rice, Cathy; University of Southampton<br>Bostock, Jennifer; Quality and Outcomes of Person-Centred Care Policy Research Unit<br>Willcox, Merlin; University of Southampton, Primary Care Research Centre<br>Little, Paul; University of Southampton, Primary Care Research Centre<br>Yardley, Lucy; University of Southampton, School of Psychology; University of Bristol, School of Psychological Science
Keywords:	COVID-19, Infection control < INFECTIOUS DISEASES, QUALITATIVE RESEARCH, Public health < INFECTIOUS DISEASES

SCHOLARONE™
Manuscripts

I, the Submitting Author has the right to grant and does grant on behalf of all authors of the Work (as defined in the below author licence), an exclusive licence and/or a non-exclusive licence for contributions from authors who are: i) UK Crown employees; ii) where BMJ has agreed a CC-BY licence shall apply, and/or iii) in accordance with the terms applicable for US Federal Government officers or employees acting as part of their official duties; on a worldwide, perpetual, irrevocable, royalty-free basis to BMJ Publishing Group Ltd ("BMJ") its licensees and where the relevant Journal is co-owned by BMJ to the co-owners of the Journal, to publish the Work in this journal and any other BMJ products and to exploit all rights, as set out in our licence.

The Submitting Author accepts and understands that any supply made under these terms is made by BMJ to the Submitting Author unless you are acting as an employee on behalf of your employer or a postgraduate student of an affiliated institution which is paying any applicable article publishing charge ("APC") for Open Access articles. Where the Submitting Author wishes to make the Work available on an Open Access basis (and intends to pay the relevant APC), the terms of reuse of such Open Access shall be governed by a Creative Commons licence – details of these licences and which Creative Commons licence will apply to this Work are set out in our licence referred to above.

Other than as permitted in any relevant BMJ Author's Self Archiving Policies, I confirm this Work has not been accepted for publication elsewhere, is not being considered for publication elsewhere and does not duplicate material already published. I confirm all authors consent to publication of this Work and authorise the granting of this licence.

For peer review only

Infection control in the home: A qualitative study exploring perceptions and experiences of adhering to protective behaviours in the home during the COVID-19 pandemic

Katherine Morton[1¶], Lauren Towler[1*¶], Julia Groot[2], Sascha Miller[1], Ben Ainsworth[2,3], James Denison-Day[1], Cathy Rice[1,4], Jennifer Bostock[5,6], Merlin Willcox[7], Paul Little[7], & Lucy Yardley[1,8]

[1]School of Psychology, University of Southampton, Southampton, United Kingdom

[2]Department of Psychology, University of Bath, Bath, United Kingdom

[3]National Institute for Health Research Biomedical Research Centre, Faculty of Medicine, University of Southampton, Southampton, United Kingdom

[4]Public Contributor, Bristol, United Kingdom

[5]Quality Safety & Outcomes Policy Research Unit, University of Kent & Oxford, Kent, United Kingdom

[6]Public contributor, London, United Kingdom

[7]Primary Care Research Centre, University of Southampton, Southampton, United Kingdom

[8]School of Psychological Science, University of Bristol, Bristol, United Kingdom

* Corresponding author

Postal address: B44 University Road, Southampton, SO17 1BJ

Email: lbt1g14@soton.ac.uk

¶ KM and LT are joint first authors.

Word count: 5649

For peer review only

Abstract

Objectives: We sought to explore people's experiences and perceptions of trying to implement infection control behaviours in the home during the COVID-19 pandemic, in order to increase our understanding of issues relevant to adherence, and to inform the adaptation of an online behavioural intervention called Germ Defence.

Participants and Design: Thirteen participants completed think-aloud interviews via phone. Data were analysed using thematic analysis. The interview analyses were triangulated with analyses of qualitative data from an open-ended online survey completed by 124 Germ Defence users.

Results: Thematic analysis generated 7 themes*: perceived risk; belief in the effectiveness of protective behaviours; acceptability of distancing and isolation; having capacity to perform the behaviours; habit forming reduces effort; having the confidence to perform the behaviours; and social norms affect motivation to engage in the behaviours.* Behaviours such as isolating and social distancing at home were identified as less acceptable than cleaning and handwashing, influenced by the need for connection and intimacy with other household members. This was especially true in the absence of symptoms and when levels of perceived risk were low. People felt more empowered when they understood that even small changes, such as spending *some* time apart, were worthwhile to reduce exposure and lessen viral load.

Conclusions: The current study provided valuable insight into the acceptability and feasibility of the protective behaviours, and how public health guidance could be incorporated into a behaviour change intervention for the public during a pandemic. The findings were used to directly inform the optimisation of the Germ Defence intervention.

Keywords: COVID-19, Infection control, Perceptions, Attitudes, Health Behaviours, Qualitative

For peer review only

STRENGTHS AND LIMITATIONS OF THIS STUDY

- To our knowledge, this is the first paper to qualitatively explore attitudes toward and experiences of performing protective behaviours within the home to prevent within-household transmission, which has been shown to be a key risk.
- Think-aloud interview data were triangulated with data from 124 survey respondents, and affinity between the two data sources was high.
- Transferability of the results is potentially limited due to the rapidly shifting nature of the pandemic, and limited representation of participants from minority ethnic groups.

INTRODUCTION

Behavioural measures have been recommended to help control the spread of the COVID-19 virus, and it is known that transmission within the home is a key risk.[1,2] However, evidence suggests that adherence to these behaviours is mixed in the UK and other affected countries.[3-5]

Germ Defence is an infection control intervention which was initially developed to target seasonal colds and flu using theoretical modelling and qualitative research, in line with the person-based approach.[6] The intervention has been updated and optimised for use by the Universities of Bristol, Bath and Southampton to help people protect themselves at home from COVID-19,[3,7] and its implementation into primary care is currently being trialled.[8] The theory of planned behaviour (TPB) was applied to identify behavioural determinants on which to base the content.[9] Leventhal's common-sense model of health and illness was used to ensure the website content attended to common perceptions and constructions of illness and infection.[10] To increase users' perceived risk, the intervention is structured using protection motivation theory (PMT) by emphasising the personal and social health consequences of contracting COVID-19.[11] Evidence suggests that TPB and PMT concepts

For peer review only

in particular explain behavioural responses during a pandemic.[12] Risk messages are followed by supportive coping messages explaining how users can reduce that risk by lowering their contact with the virus. The language used on the website is in line with self-determination theory to increase users' motivation to carry out the behaviours.[13] Intervention content, design and structure was informed by qualitative think-aloud interviews with the general public.[14]

This study sought to explore experiences and perceptions of performing protective behaviours at home in order to identify possible barriers and facilitators, and develop an understanding of how these behaviours are influenced by perceptions. This forms part of the person-based approach to adaptation and optimisation of the Germ Defence intervention for COVID-19.[15]

METHOD

Participants

Inclusion criteria were those over the age of 18, able to access the Germ Defence website and able to give informed consent. Participants were recruited via two main routes: users of the Germ Defence website (n=7) who registered their interest, and non-users accessed through social media and newsletters sent out by organisations and community groups (n=6). Interview participants were purposively sampled by factors such as age, gender, education level, risk status and experience of COVID-19 to maximise diversity. Users of Germ Defence who volunteered to participate in research but were not purposively sampled for an interview were invited to complete a short questionnaire instead.

Survey

A total of 124 website users completed the qualitative survey (n = 545 invited, 23% response rate). Most participants were over 60 years old, reported being at increased risk from

For peer review only

COVID-19, and White British. Table 1 shows the demographic details of the survey respondents.

Table 1. Survey respondent demographics

		N	%
Age	26-40	2	1.6
	41-60	37	29.8
	61-70	41	33.1
	70+	31	25
	Missing	13	10.5
Experience with COVID-19			
	I am at increased risk	50	40.3
	Someone I live with is at increased risk	19	15.3
	I think I've had COVID-19	7	5.6
	I think someone I live with has had COVID-19	1	0.8
	None of the above/No experience	33	26.6
	Unassigned	14	11.3
Ethnicity	White British	101	81.5
	White Irish	1	0.8
	White European	2	1.6

For peer review only

	White Canadian	2	1.6
	Black British	1	0.8
	Black African	1	0.8
	British Chinese	1	0.8
	Missing	15	12.1
Education level	Pre-secondary school	1	0.8
	Secondary School	43	34.7
	Undergraduate	38	30.6
	Postgraduate	28	22.6
	Missing	14	11.3

Interviews

Table 2 shows the demographic details of the 13 interview participants. The mean interview length was 79 minutes (range 60-104 minutes). Most participants lived with at least one other person, and 7 participants felt that either they or a household member was at increased risk should they contract the virus.

Table 2. Interviewee demographics.

ID	Sex	Age	Date interviewed	Household members
1	F	61-70	08/06/2020	Lives with spouse and teenage children
2	F	61-70	11/06/2020	Lives with husband with cancer
3	F	41-60	12/06/2020	Lives with teenage children
4	F	61-70	29/06/2020	Lives alone

For peer review only

5	F	41-60	01/07/2020	Lives with older parents with comorbidities, spouse, and teenage child
6	F	61-70	03/07/2020	Lives with partner
7	F	41-60	07/07/2020	Lives with spouse and adult son
8	F	41-60	16/07/2020	Lives alone
9	M	18-25	23/07/2020	Lives with parents and sister
10	M	26-40	10/09/2020	Lives with partner
11	F	61-70	21/09/2020	Lives with husband with comorbidities
12	F	26-40	28/09/2020	Lives with partner
13	F	26-40	05/11/2020	Lives with partner

For peer review only

Measures

Demographics

Potential participants were asked to complete an online survey to determine age, gender, experience of COVID-19, education, household size, postcode to inform Index of Multiple Deprivation, and ethnicity. Finally, contact information was collected to enable a researcher to invite the potential participant to interview or to complete the survey.

Topic Guide

Think-aloud semi-structured interviews[16,17] were conducted by three female interviewers (LT, KM and JG), in which the participants provided feedback on each page of the online intervention (https://www.germdefence.org/) to provide detailed insights into their perceptions of the content.[3] At the beginning of the interviews, participants were asked a series of questions pertaining to their general perceptions of COVID-19 and protecting

themselves at home (e.g "Can you tell me how you feel about the coronavirus at the moment?"). Then, the participants used the website and the researcher asked them what they thought of the content on each page. All interviewers were researchers within the field of health psychology. Prompts or follow-up questions typically pertained to attitudes toward the behavioural information and determinants of engagement and adherence. At the close of the interview, a series of general questions were asked about their overall views of the Germ Defence website.

Survey

The survey featured four open-ended questions in addition to closed demographics questions. The survey aimed to gather participants' thoughts on the protective behaviours suggested on the website such as, "How do you feel about following the suggestions on Germ Defence?" and "What did you not like about the Germ Defence advice?"

Procedure

Those who accessed the Germ Defence website and completed at least one section saw a pop-up banner asking if they might be interested in taking part in research to help improve the website. If they indicated they wished to take part in research they were asked to complete the online demographic questions hosted by Qualtrics to inform purposive sampling. In addition, adverts inviting people to take part in a telephone interview about a website designed to help keep them and their household safe from coronavirus were posted on social media, with a link to the purposive sampling questions. Ethical approval was granted by the University of Southampton Psychology Ethics Committee (ID: 56445).

Interviews

Participants were purposively selected by the research team and sent a link to the information sheet and consent form, which was completed online. Interviews were conducted by

For peer review only

telephone, due to the pandemic. The audio recording began once consent was verbally reaffirmed. At the close of the interview, participants were offered a summary of the results, and were thanked with an Amazon voucher. The interviews took place during a period of rapidly changing guidelines in the UK, from 8th June to 5th November 2020, most whilst the R-rate was relatively low, and restrictions were soon to be (or had already been) lifted.

Survey

A total of 545 respondents were invited to complete the survey over three separate mail-outs: the first on 19th June 2020 (n=150); the second on 10th July 2020 (n=103); and the third on 24th July 2020 (n=292). The email contained a link to the survey, which began with a participant information sheet and consent form. For context, the first mail-out occurred during the first lockdown, which was lifted on 4th July 2020, but wearing face-coverings inside shops only became compulsory on the date of the final mail-out; 24th July 2020.

Patient and Public Involvement (PPI)

As Germ Defence is available to the general public, PPI was integral to its development. Two public contributors (CR and JB) on our stakeholder panel participated in weekly meetings which informed the optimisation of the intervention, and worked with us to identify potential issues in the behavioural messages of the intervention and update the intervention content in line with feedback. The conceptualisation, measures, recruitment strategy and dissemination of the current study was informed by open discussion with these members. For example, the public contributors reviewed the interview topic guide and assisted in identifying which organisations to target during the recruitment process. In particular, the public contributors provided considerable assistance in ensuring that the study materials and study invitations were easy to understand and free of jargon. In addition, users of the Germ Defence website could volunteer for one-off PPI engagement to give feedback on the website content, either

For Peer review only

by taking part in a survey or an interview. Further detail on PPI in the development and optimisation of Germ Defence has been reported elsewhere.[15]

Data Analysis

Interviews

Data were analysed using inductive thematic analysis to openly explore the barriers and facilitators that were important to people.[18,19] Due to the need for rapid analysis and dissemination of initial findings, the first set of transcripts were split between two researchers (n = 6 transcripts analysed by KM and n = 3 transcripts by LT). The researchers independently read their transcripts thoroughly to first familiarise themselves with the data. Data were then coded inductively by unit of meaning using NVivo, keeping the core aims of the study in mind (barriers and facilitators to, and perceptions of, infection control behaviours in the home). After the first nine interviews had been coded, the researchers met and compared their coding manuals, discussing each code and theme in detail and generating a final agreed coding manual to unite their coding. This involved revisiting the raw data to confirm shared and consistent understanding of how the codes and themes were being used. The coding manual was then used by LT to code the remaining four interviews, and where necessary new codes were added and existing codes were further refined, although these amendments were only minor. LT double-checked the earlier transcripts to ensure the revised coding manual was consistently applied across the data, and the researchers met again to confirm agreement on the final coding manual.

Survey

The results from the survey were used to triangulate the interview data, to validate and enrich our findings. Responses to the four open-ended questions were coded inductively and categorised thematically separately from the interview data analysis. Inductive coding was

For peer review only

deemed most appropriate, as the researchers intended to triangulate for complementarity, rather than convergence, to ensure that any unique perspectives gathered from the survey data were attended to. The resulting categories were then mapped onto the themes generated from the interview data to assess their fit with these themes, whether any new themes or subthemes were present in the survey data, and to what extent the survey data provided further nuance to the existing themes.

RESULTS

The researchers generated 7 key themes from the interview data related to perceived barriers and facilitators to engaging with infection control behaviours in the home. These were: *perceived risk*; *belief in the effectiveness of protective behaviours*; *acceptability of distancing and isolation*; *having capacity to perform the behaviours*; *habit forming reduces effort*; *confidence in how to perform the behaviours*; and *social norms affect motivation to engage in the behaviours*. See Online Resource 1 for the coding manual. Extracts from the interview data are delineated by the abbreviation 'int'.

For the survey, most respondents felt positively about the protective behaviours recommended on the Germ Defence website. The themes identified from the survey data mapped well onto the interview analysis, with particularly strong congruence to *confidence in how to perform the behaviours.* The survey findings are discussed alongside the interview data within the themes which they mapped onto. Extracts from the survey data are delineated by the letter 's'.

Perceived risk

Germ Defence encourages users to evaluate their own level of risk and which actions they feel are appropriate for them based on this level of risk, to enable users to focus on the behaviours and advice they deem the most personally relevant. For more detail on the

For peer review only

intervention content and how we tailored it for perceived risk, see other publications from the project.[3,20] Participants' assessments of their level of risk played a major role in their willingness to engage in the protective behaviours, particularly those seen as more 'extreme' such as social distancing from other household members. Those who perceived that the virus is likely to enter their home, and/or that household members are at risk of becoming seriously unwell were generally highly motivated to engage with the behaviours.

Current levels of virus in circulation

Information about the current actual risk of infection was important for some people to help make decisions about performing difficult behaviours. For example, a mother justified her reluctance to follow social distancing guidance in the home in terms of the lower perceived necessity to do this at the moment.

> *"There is that sort of hope that, as there is I think known to be that much less of the virus out there generally at the moment… although we're still taking all the precautions, there is that hopefulness that the risk is less now than it was back in March."* (int 3)

> *"I didn't follow the stricter suggestions such as using disinfectant in the home, as we're low risk and the area we live in has very low numbers of cases."* (s71)

Perceived likelihood of virus entering the home

Some participants were concerned about those in the household bringing the virus home if they needed to leave for work. This was influenced by how much mixing the person was doing outside the home, and the perceived severity of the consequences if someone in the household became ill.

For peer review only

"They said only one person is allowed out during the lockdown. So it was my husband… I was worried, because I'm the one who does the cooking and things, that I would pass it on to my parents if he caught it." (int 5)

Having people from outside the household in the home was felt to be a significant risk. Participants were generally highly motivated to engage in the protective behaviours when visitors were present.

"*I had a workman come in and he had to look at – because my heating's gone – and I was having a heart attack with him touching anything. So I was going round spraying everything with bleach like a maniac, even the carpet. So what are you meant to do if you've got workmen. I made him wear a mask, I made him wear gloves.*" (int 5)

"*Well I'm not going in anybody's house, and I'm not having anybody in my house…My house is my safe haven."* (int 4)

Perceived risk of severe consequences to health

People's perceived risk of severe illness or death from the virus was influenced by co-morbidities (such as cancer, COPD, asthma, and high blood pressure), old age, ethnicity, and being an inter-generational household.

"When you've taken a decision to tell your parents to come and live with you, and then you're reading stuff about intergenerational households, it's a much higher risk…" (int 5)

One participant described how she decided to shield with her husband to protect him, despite not being classed as vulnerable herself.

For peer review only

> *"I would just be so petrified I was going to give him something… I feel less… kind of imprisoned in a way, by shielding myself with him, than going out into the so-called freedom, but then coming back and being petrified I'll kill him."* (int 2)

Another described how one of the younger members of her household felt he didn't need to worry about the virus because of his age, and he perceived that only those at increased risk needed to be concerned.

> *"Our young man thinks that the only people that you should be worried about are people that are at increased risk, should they catch it. Not everybody else. Do you know what I mean, it's like, oh well, it doesn't matter because they're fine, my friends are fine."* (int 1)

Belief in the effectiveness of the protective behaviours

The perceived effectiveness of behaviours appeared to influence participants' willingness to engage with them. Participants identified an important caveat: the virus could spread within the home before symptoms present, meaning that protective behaviours could be viewed as pointless unless performed consistently. However, perceiving viral load to be a factor in viral transmission seemed to mitigate this, and these participants felt empowered to enact small changes around their home to reduce their risk.

Perceived value of cleaning

Most participants were already very aware of cleaning and washing hands and felt these were important. However, cleaning was sometimes associated with being paranoid and fearful, and some participants were keen to explain they weren't paranoid about the level of cleaning they do, whilst others described how the virus has made them feel paranoid about cleaning.

For peer review only

“Careful but not paranoid, yeah. I don’t wash my keys in soapy water, and I don’t regularly wash my car. We just wash and hand gel our hands after we’ve been somewhere that’s in the car, when we get back into it.” (int 7)

“at the beginning I was cleaning constantly. I still am…. And then I’m spraying, down the surfaces with disinfectant, because I’m worried about this transference. Okay, you’ve just touched it, so you’ve put it down. So that now gets onto that surface, if somebody in the meantime touches that surface, it then carries on and then goes onto another surface. That’s what I’m on about, with the paranoia.” (int 5)

Perceived value of wearing a face-covering

People’s willingness to wear a face-covering was strongly influenced by perceptions of effectiveness, although the focus was on wearing them outside the home. Most of the interviews took place prior to the mandatory use of face-coverings in the UK, and there was some uncertainty and variance within the public discourse regarding their effectiveness at the time. These sentiments were reflected by our participants. Some people had read information from other countries which convinced them that face-coverings were an effective way to prevent transmission, and one participant emphasised how she believed face-coverings were important for protecting others more than yourself, whereas a few remained unconvinced and wanted more evidence.

“*I might wear a mask, like I told you, I need to do more research on that.*” (int 6)

Reasons offered for why masks might be ineffective included lack of filters, the mask causing infection due to dampness from breath, and people touching their face. Furthermore, at the start of the pandemic and during the time in which most of the data collection took place, infection control strategies (including Germ Defence) placed a strong focus on surface transmission. As the pandemic progressed, the focus has shifted to airborne transmission,

For peer review only

particularly the importance of ventilation. However, since manual transmission remains a potential transmission pathway within the home, Germ Defence was altered to additionally emphasise airborne transmission, rather than reduce the emphasis on handwashing and surface transmission. For more information on the advice given in Germ Defence and how this has changed during the progression of the pandemic based on PHE, PPI and stakeholder input, see further publications from the project.[15,20] This could explain why our participants reported stronger beliefs in the value of cleaning surfaces over face-covering and ventilation.

Barrier: Virus is likely to spread before you know you're ill

Some people were uncertain whether it would be achievable to prevent the virus spreading in the home.

> *"I think I probably still am, to a certain extent, sceptical about whether we would be able to get a virus come into this home and avoid spreading it between us."* (int 3)

People were concerned that the virus would already have spread by the time they socially distanced or self-isolated, making it pointless unless done continually.

> *"If at any stage I started to feel ill, which is probably then too late, because I probably would've then spread it to them, I could've potentially spread it to them by then anyway, I would then take myself to my room."* (int 3)

Facilitator: Reducing all or nothing thinking

People were more likely to perceive protective behaviours as effective and worthwhile when they perceived catching the virus as a continuum based on how much viral load you are exposed to, rather than you either catch it or not.

For peer review only

"I use antibacterial wipes on just about all the shopping that comes into the house as well, when it's delivered, just as a precaution. Because I think it's safer if you do get the virus that it's as small as possible." (int 7)

This was empowering as it helped people feel that small changes can still make a difference.

"I am sitting here thinking, if I turned the table the other way around, we could actually sit further apart from each other at the table, which might be one small thing." (int 3)

Survey participants also highlighted the importance of balancing behaviours in accordance with personal risk level and perceived negative impact of the behaviour (e.g. social distancing negatively impacting wellbeing), linking in with the perceived risk theme.

"*It might not be good to be keeping them* [children] *at 2 m away for their development or mental health. Need more nuances about balancing risk against looking after child development."* (s18)

Acceptability of distancing and isolation

Social distancing and isolation behaviours were presented on the Germ Defence website as recommended for higher risk individuals, but also as useful ideas for lower risk households to help reduce risk whenever it was deemed necessary. Spending time together was perceived as integral to the wellbeing of the household, but some participants described small changes they had made to help maintain intimacy while social distancing or self-isolating. Social distancing and self-isolation were seen by some as only acceptable for short periods of time when symptoms were present.

Barrier: Importance of time together

For peer review only

The idea of self-isolating within the home was quite daunting for people and there was some concern about the effect upon mental wellbeing. Experiences of intimacy with partners and family members was generally judged to be of higher importance than reducing the risk of virus transmission when no symptoms were present, even when some members of the household were high risk.

> *"I don't think I could cut down on the amount of time I spend with other people, because they'll get lonely…"* (int 5)

> *"Because to a ninety-five-year-old a kiss is more important than worrying about whether or not you're going to die of a virus."* (int 11)

Some people described spending some time on their own during the day, but the evening meal was often regarded as an important time to spend together.

> *"The evening meals are nice… that's the one thing where we don't really take any precaution with the family, just because we all sit around the dinner table. But that is a nice part of the day, really, so in that respect it's quite good for everyone's mental health."* (int 9)

One couple found the idea of eating separately with the at-risk individual in his room as completely unacceptable:

> *"I think the guidance said something awful, like he should stay in his own room and be, you know, deliver his food to him like he was a kind of caged animal."* (int 2)

Some people perceived social distancing as acceptable for short periods of time if someone is ill, but not as something to do indefinitely as a preventative measure.

> *"Is that something I would have to do all the time, every day of my life? And then that feels completely… I wouldn't feel that there was much quality of life if I had to… if*

For peer review only

I'm living in the same house as my children at the moment but I couldn't hug them or sit near them or… It's something I could see potentially doing if it was for a limited period, but it just feels impossible sort of long-term." (int 3)

Facilitator: Ways of maintaining (distanced) intimacy

Some participants had made changes at home to enable social distancing, and they described how they managed to maintain some feelings of intimacy. Small changes to furniture arrangements or daily routines, the use of technology, and contact which was perceived to be low risk were seen as effective ways to engage with the protective behaviours without completely sacrificing intimacy and connectedness:

"I added on an extra table in the dining room, so that I could keep a metre from him when we're eating, even though it's joined eating." (int 5)

"We have a bit of a dry cuddle, like I go over his shoulders, but I don't breathe on him and he doesn't breathe on me. So we're kind of on board with it, you know?" (int 2)

"In the morning, I go and wake him up and say, "Oh, I'm getting up now for work," and he goes down and makes me a cup of tea, just because we kind of like to have that... But he will deliver it to my dressing table and then I'll pick it up and take it back to bed. It's kind of trying to keep that intimacy, but without actually sharing everything." (int 2)

Having capacity to perform protective behaviours

This theme explores participants' perceptions of the practical factors which affect their capacity to perform the suggested behaviours. Having sufficient space was an important

For peer review only

factor in how feasible it was for people to socially distance and self-isolate. Those who lived in smaller spaces generally found the idea of social distancing unfeasible.

> "*I'm guessing this applies to people in like houses more than just like one room, 'cause I currently live in a flat, a one bed flat with my partner, so it's kind of impossible for us to have one room in our home to be just for us.*" (int 12)

Some people found it challenging trying to implement house rules for others to follow during the pandemic. Hand-washing was a particular behaviour mentioned that participants tried to persuade partners and children to do, or checked whether they had done, which was identified as a source of tension.

> *"But when he comes home, I tell him to wash his hands, and every time he gets home, I'm always, "Have you washed your hands?"* (int 6)

> *"I will just keep reminding him, all the time, to wash his hands. And he'll say, "I've done it." You say, "No you haven't. The sink's not wet." And, "well I did it. I did do it, I did it when I got to my...' Because he's a sink in his room, "I did it when I got to my room" which we know is not necessarily the case. So it's… it's tricky, but we're trying to keep on the case."* (int 1)

Habit forming reduces effort

When discussing the effort involved in performing the protective behaviours, participants typically discussed how well they integrated with their current behaviours and routines. Some participants described how some protective behaviours, such as cleaning, regular hand-washing and not sharing towels, had already been the norm for them before the pandemic, which helped them to adhere.

For peer review only

"I found that they are things that I have always done, throughout my life, because I was taught to as a child." (int 4)

Social distancing was also facilitated in some households with teenage children, who were described as spending a lot of time in their rooms anyway. Additionally, despite an initial negative reaction to social distancing, some described how working from home meant that they were spending most of their time away from other household members.

"*I'm looking at it going, 'Really? You think this is a rational thing to do?' Like I mean, I do sit in a room on my own for most of the day, funnily, because I work from home, as does my partner, and you know, it… she'd irritate me if she was on calls and vice versa, so yeah, we do sit separately.*" (int 10)

Where new behaviours had become habits for our participants, they perceived less effort involved in performing the behaviours.

"I think they're definitely becoming habits now. I mean, it is… still is harder than it used to be, because I never would've done that before. But it is more normal now." (int 3)

Others who were being extremely careful about cleaning found it could be quite effortful and fatiguing. It seemed that participants living with people at increased risk were more likely to find the constant cleaning demanding.

"It feels like it's a constant state of vigilance. It's very high intensity, that level of concentration all the time, not to lapse." (int 5)

Confidence in how to perform the behaviours

For peer review only

Both interview and survey participants identified that they wanted clear and consistent practical information on what to do. Inconsistent information seemed to undermine people's confidence in their ability to perform the behaviours and reduce their risk.

> "*It's easier now than when it first started…I feel like the mask guidance just came out of nowhere, so one minute they're telling us that they don't have any scientific evidence, and the next minute it's, 'from the 30th you have to wear masks,'… it was just strange…*" (int 12)

However, both interview and survey participants felt more confident in their ability to engage with and perform the behaviours when they felt well informed and affirmed by those who they perceived to be experts.

> "*Knowing the advice came from trusted source gave me confidence and so helped to avoid fear/anxiety overwhelming.*" (s81)

When participants felt that they were doing the 'right' thing, they felt empowered and motivated to continue. The Germ Defence website encourages users to plan how much they intend to engage with the behaviours going forward. If their plans show that their adherence will improve, they are given positive reinforcement:

> "*It's quite validating…I've reconsidered what I've been doing and now I'm going to make the steps, and I feel quite empowered.*" (int 12)

Social norms affect motivation to engage in the behaviours

Some participants discussed how they felt demotivated to engage in the behaviours when they perceived others were disregarding infection control advice. These participants felt that protective behaviours were simply "not worth the effort" when others were not playing their part.

For peer review only

"I feel a little bit disenchanted by the whole thing, because you know, I've done things properly, … I didn't leave the house for… three months. And even when it was relaxed I didn't, and yet I still have to watch my neighbour, who's seventy-five, going out for a drive every single day during lockdown, and that is difficult to take. So it was a bit like 'actually … why am I doing my bit here, when everyone else isn't?'" (int 10)

DISCUSSION

These findings show how people conceptualise the risk of catching and transmitting COVID-19, and use this as a rationale for their behaviour at home. Perceived risk increased willingness to adhere to protective behaviours, as did perceived effectiveness of the behaviours. Cleaning and handwashing were widely perceived to be effective and acceptable, although some participants described how other members of their household were less adherent to these behaviours which could cause anxiety. Participants also found the protective behaviours easier if they fit well with their usual routine, suggesting that linking the new behaviours to more ingrained habits could increase adherence.

Behaviours such as spending time in separate rooms at home and keeping two metres apart were less acceptable, especially as preventative measures to follow even when no-one in the household has any symptoms (although this was only suggested for higher risk households). Our participants generally felt that a lack of physical and emotional closeness with their household members was too much of a sacrifice to engage in social distancing regularly, even when the household was identified as high risk. Additionally, since our participants tended to find the behaviours easiest to adhere to when they fit well with their usual routine and when they formed a habit, it could be that these particular behaviours are seen as too different from their typical way of life. Finally, awareness of the concept of viral load helped people feel

For peer review only

more empowered as they understood that even small changes, such as spending *some* time apart, were worthwhile.

In addition to these more novel concepts, there was also some congruency between the current findings and the previous research into adherence to infection control behaviours during a pandemic. Our participants also indicated that they felt some concern about being perceived as paranoid by those around them (within the subtheme; *perceived value of cleaning)*, indicating that there may still be negative social connotations surrounding hygiene practices.[14] Our findings regarding the need for emotional connection and intimacy provide support for the findings from recent qualitative research into the impact of COVID-19 and adherence to government guidance. Some may only partially adhere to the behaviours due to the need for and cultural importance of social contact, and some reported feelings of loss and grief over the loss of social interaction during lockdown.[21-23] Additionally, the need for clarity and consistency in government and public health guidance has also been highlighted in other studies as important in aiding the public to adhere to infection control behaviours.[21,23,24]

Furthermore, perceived risk levels and greater belief in the effectiveness of the protective behaviours were found to be important predictors of behaviour in a previous review.[12] Finally, our participants expressed some concern and awareness that transmission to other household members may well have occurred by the time that symptoms present, supporting previous qualitative research into the public's opinions of the need for separate accommodation for at-risk individuals during the COVID-19 pandemic.[25] This indicates a need for preventative educational interventions so that the public are equipped to act as soon as they feasibly can.

Strengths and Limitations

For peer review only

Triangulation of the think-aloud data with open-ended survey data revealed very high affinity between the two datasets, suggesting that the themes identified are valid and robust. However, the transferability of our results should still be treated with some caution due to the rapidly changing nature of the pandemic and government guidance, and because our sample may not represent the views of the general population. Half of our interview participants were Germ Defence users, recruited from the website itself after receiving the intervention. It is therefore likely that they were more engaged and motivated than the general population since they sought out the information for themselves and subsequently volunteered to participate in research. The data gathered from non-website users did not differ substantially from the website users' experiences, discussing largely similar themes and concepts – although these volunteers are also likely to have an interest in reducing transmission. Our survey sample was also predominantly White Caucasian, and no interview participants identified themselves as belonging to Black, Asian and minority ethnic (BAME) groups. While efforts were made to purposively sample for greater diversity, the need for rapid data collection to inform the optimisation of the intervention limited our recruitment options. However, as noted above, some similar concepts to the current findings were found in a recent interview study which focused on members of low income and ethnic minority households.[22]

Conclusions and Implications

Our findings have several implications for behavioural interventions and public health guidelines during a pandemic. These findings have shown that the public may be unwilling to adhere to the protective behaviours indefinitely if they perceive the risk to be low, so it is important that behavioural guidelines encourage accurate perceptions of personal risk level, and highlight that enacting even small changes would still be worthwhile for reducing risk. People understood the concept of viral load and found this a helpful rationale for making small changes which could be maintained over time. Furthermore, the perceived negative

For peer review only

impact of social distancing and isolation on mental wellbeing within the home seems to be a major sticking point in terms of the public's willingness to adhere. Behavioural interventions which offer practical suggestions for how intimacy could be maintained whilst socially distancing could reassure the public that they could reduce the negative impact on their wellbeing whilst engaging with protective behaviours, at least some of the time.

FUNDING STATEMENT

The study was funded by United Kingdom Research and Innovation Medical Research Council (UKRI MRC, https://mrc.ukri.org/) Rapid Response Call: UKRI CV220-009. The Germ Defence intervention was hosted by the Lifeguide Team, supported by the NIHR Biomedical Research Centre, University of Southampton. LY is a National Institute for Health Research (NIHR) Senior Investigator and theme lead for University of Southampton Biomedical Research Centre. LY is affiliated to the National Institute for Health Research Health Protection Research Unit (NIHR HPRU) in Behavioural Science and Evaluation of Interventions at the University of Bristol in partnership with Public Health England (PHE). MW is an NIHR Academic Clinical Lecturer, under grant CL-2016-26-005.The views expressed are those of the author(s) and not necessarily those of the NHS, the NIHR, the Department of Health or Public Health England. The funders had no role in study design, data collection and analysis, decision to publish, or preparation of the manuscript.

COMPETING INTERESTS STATEMENT

The authors declare that no competing interests exist.

DATA SHARING STATEMENT

Data cannot be shared publicly because our participants did not consent to the publication of full transcripts. Only extracts are made available to those outside of the study research team. A coding manual containing additional extracts has been supplied as an online resource.

For peer review only

AUTHOR CONTRIBUTIONS

Katherine Morton

Conceptualization, Formal Analysis, Investigation, Methodology, Project Administration, Data Curation, Resources, Validation, Writing – Original Draft Preparation, Writing – Review & Editing

Lauren Towler

Conceptualization, Formal Analysis, Investigation, Methodology, Project Administration, Data Curation, Resources, Validation, Writing – Original Draft Preparation, Writing – Review & Editing

Julia Groot

Investigation, Project Administration, Writing – Review & Editing

Sascha Miller

Conceptualization, Resources, Writing – Review & Editing

Ben Ainsworth

Conceptualization, Funding Acquisition, Resources, Writing – Review & Editing

James Denison-Day

Software, Resources, Writing – Review & Editing

Cathy Rice

Resources, Writing – Review & Editing

Jennifer Bostock

Resources, Writing – Review & Editing

For peer review only

Merlin Willcox

Resources, Writing – Review & Editing

Paul Little

Conceptualization, Funding Acquisition, Resources, Writing – Review & Editing

Lucy Yardley

Conceptualization, Funding Acquisition, Resources, Writing – Review & Editing

ACKNOWLEDGEMENTS

We would like to thank our voluntary research assistants, Amina Khan and Lara Rosa, who led engagement with social media and charities for study recruitment.

REFERENCES

1. Wang Z, Ma W, Zheng X, et al. Household transmission of SARS-CoV-2. *J Infect*. 2020;81(1):179-82. doi: 10.1016/j.jinf.2020.03.040
2. Wang Y, Tian H, Zhang L, et al. Reduction of secondary transmission of SARS-CoV-2 in households by face mask use, disinfection and social distancing: a cohort study in Beijing, China. *BMJ Glob Heal*. 2020;5(5):e002794. doi:10.1136/bmjgh-2020-002794
3. Ainsworth B, Miller S, Denison-Day J, et al. Infection Control Behavior at Home During the COVID-19 Pandemic: Observational Study of a Web-Based Behavioral Intervention (Germ Defence). *J Med Internet Res*. 2021;23(2):e22197. doi: 10.2196/22197
4. Bodas M, Peleg K. Self-isolation compliance in the COVID-19 era influenced by compensation: Findings from a recent survey in Israel. *Health Aff*. 2020;39(6):936-41. doi: 10.1377/hlthaff.2020.00382

For peer review only

5. Machida M, Nakamura I, Saito R, et al. Adoption of personal protective measures by ordinary citizens during the COVID-19 outbreak in Japan. *Int J Infect Dis*. 2020;94:139-144. doi: 10.1016/j.ijid.2020.04.014

6. Yardley L, Morrison L, Bradbury K, et al. The person-based approach to intervention development: Application to digital health-related behavior change interventions. *J Med Internet Res*. 2015;17(1):e30. doi: 10.2196/jmir.4055

7. Ainsworth B, Steele M, Stuart B, et al. Using an analysis of behavior change to inform effective digital intervention design: how did the PRIMIT website change hand hygiene behavior across 8993 users? *Ann Behav Med*. 2016;51(3):423-431. doi: 10.1007/s12160-016-9866-9

8. Horwood J, Chalder M, Ainsworth B, et al. Primary Care implementation of Germ Defence, a digital behaviour change intervention to improve household infection control during the COVID-19 pandemic: A structured summary of a study protocol for a randomised controlled trial. *Trials*. 2021;22(263). https://doi.org/10.1186/s13063-021-05188-7

9. Ajzen I. The theory of planned behavior. *Organ Behav Hum Decis Process*. 1991;50:179–211. doi: 10.1016/0749-5978(91)90020-T

10. Leventhal HA, Brissette I, Leventhal EA. The common-sense model of self-regulation of health and illness. In: Cameron LD, Levanthal H, editors. The self-regulation of health and illness behaviour. London: Routledge 2003:42–65.

11. Rippetoe PA, Rogers RW. Effects of components of protection motivation theory on adaptive and maladaptive coping with a health threat. *J Pers Soc Psychol*. 1987;52(3). doi: 10.1037//0022-3514.52.3.596

For peer review only

12. Bish A, Michie S. Demographic and attitudinal determinants of protective behaviours during a pandemic: a review. *Br J Health Psychol*. 2010;15(4):797–824. https://doi.org/10.1348/135910710X485826

13. Deci EL, Ryan RM. The "what" and the "why" of goal pursuit: Human needs and the self-determination of behavior. *Psychol Inq*. 1998;11:227–268. doi: 10.1207/S15327965PLI1104_01

14. Yardley L, Miller S, Teasdale E, et al. (2011). Using mixed methods to design a web-based behavioural intervention to reduce transmission of colds and flu. *J Health Psychol*. 2011;16(2):353–364. doi: 10.1177/1359105310377538

15. Morton K, Ainsworth B, Miller S, et al. Adapting behavioural interventions for a changing public health context: A worked example of implementing a digital intervention during a global pandemic using rapid optimisation methods. *Front Public Health*. 2021;9:668197. doi:10.3389/fpubh.2021.668197

16. Cotton D, Gresty K. Reflecting on the think-aloud method for evaluating e-learning. *Br J Educ Technol*. 2006;37(1):45-54. doi: 10.1111/j.1467-8535.2005.00521.x

17. Morrison L, Muller I, Yardley L, et al. The person-based approach to planning, optimising, evaluating and implementing behavioural health interventions. *Eur Heal Psychol*. 2018;20(3);464-469.

18. Braun V, Clarke V. Using thematic analysis in psychology. *Qual Res Psychol*. 2006;3(2):77-101. doi:10.1191/1478088706qp063oa

19. Clarke V, Braun V. Successful Qualitative Research: A Practical Guide for Beginners. London: Sage Publishing 2013.

20. Miller S, Denison-Day J. Applying the Person Based Approach for rapid optimisation of a digital intervention during a pandemic: adapting Germ Defence for COVID-19. Department of Psychology, University of Southampton. Forthcoming 2021.

21. Alipour F, Arshi M, Ahmadi S, et al. Psychosocial challenges and concerns of COVID-19: A qualitative study in Iran. *Health*. 2020;1363459320976752. https://doi.org/10.1177/1363459320976752

22. Denford S, Morton KS, Lambert H, et al. Understanding patterns of adherence to COVID-19 mitigation measures: a qualitative interview study. *J Public Health*. 2021;fdab005. doi: https://doi.org/10.1093/pubmed/fdab005

23. Williams SN, Armitage CJ, Tampe T, et al. Public perceptions and experiences of social distancing and social isolation during the COVID-19 pandemic: a UK-based focus group study. *BMJ Open*. 2020;10(7):e039334. doi: 10.1136/bmjopen-2020-039334.

24. Benham JL, Lang R, Kovacs Burns K, et al. Attitudes, current behaviours and barriers to public health measures that reduce COVID-19 transmission: A qualitative study to inform public health messaging. *PLOS ONE*. 2021;16(2):e0246941. https://doi.org/10.1371/journal.pone.0246941

25. Denford S, Morton KS, Horwood J, et al. Preventing within household transmission of COVID-19: Is the provision of accommodation feasible and acceptable? MedRxiv 2020.08.20.20176529 [Preprint]. September 07, 2020 [cited 2021 Feb 9] https://doi.org/10.1101/2020.08.20.20176529

For peer review only

Online Resource 1

Coding Manual

Theme	Subthemes	Definition	Example quote
**Perceived risk**	Current levels of virus in circulation	Weighing up perceived risk of virus based on current infection rates	*"I want to know is it safe for me to go out? You know, what's the transmission rate where I live?"* (int 6)
	Perceived likelihood of virus entering the home	Likelihood of a household member or someone outside the home bringing the virus into their home	*"the biggest concern is just bringing it in from outside, but I'm not doing particularly too much to risk that at the moment, I wouldn't say".* (int 9)
	Perceived risk of severe consequences to health	Considering how vulnerable household members are to becoming severely ill from the virus	*"I'm fifty-three, going on fifty-four, and the age group is looking not so brilliant now, when I was looking in press reports. I know they said over seventy. I'm pretty fit, which is good. The only problem is, I have high blood pressure, and I'm on medication for that".* (int 5)
**Belief in the effectiveness of the protective behaviours**	Perceived value of cleaning	Perceived need and motivation to clean surfaces and hands based on perceived effectiveness	*"I mean, I always washed my hands a lot, obviously, working in a tea room, so I had to wash my hands all the time anyway. So I obviously now wash them for longer, and more carefully. And I also am very, very, very conscious now of whether I'm touching my face or… things like that."* (int 3)
	Perceived value of face-coverings	Perceived need and motivation to wear	*"The thing with the face coverings is, they haven't got any filters in them, these cloth ones…. And I think it*

For peer review only

		face-covering based on perceive effectiveness	*could be more infectious, because it'll get wet with your breathing. And then it's no good to anyone".* (int 6)
	Barrier: Virus is likely to spread before you know you're ill	Belief in the potential to contain the virus if it enters the home	*"do everything in my power to prevent getting it, by assuming that I haven't got it, so wearing a mask in the house, keeping two metres, trying to keep the person that's got it in the household in a separate room"* (int 9)
	Facilitator: Reducing all or nothing thinking	Perception that it is worth reducing exposure to minimise viral load, and that even small changes can make a difference	*"the less we get in touch- the less we have contact with the virus, the safer we will be"* (int 7)
**Acceptability of distancing and isolation**	Barrier: Importance of time together	Includes concerns about own or others' mental well-being if spending time apart, and the value placed on time spent together.	*"I'm worried more about like the mental health of the other people. So although we're very careful, and not mixing. So I don't think I could cut down on the amount of time I spend with other people, because they'll get lonely"* (int 5)<br><br>*"Staying 2m from my 11yr old means me pushing him away and not sharing our only front room seating with him."* (s18)
	Facilitator: Ways of maintaining (distanced) intimacy	Finding ways to maintain emotional intimacy when social distancing or self-isolating	*"We have, I had a bit of, as I said, a bit of a dry cuddle, like I go over his shoulders, but I don't breathe on him and he doesn't breathe on me. So*

For peer review only

			we're kind of on board with it, you know?" (int 2)
**Having capacity to perform protective behaviours**		Factors which reduce a person's capacity to engage with and perform the behaviours. For example; having enough space, or not, in your house to either socially distance or self-isolate. Includes descriptions of encouraging others to adhere to protective behaviours, or the challenges of trying to influence others.	*"My son sits in one settee and my husband and I sit in the other. And that… it doesn't protect us all, we're not all sitting on our own sofa, but who has three sofas in their room? So… we do what we can".* (int 7) *"I will just keep reminding him, all the time, to wash his hands. And he'll say, "I've done it." You say, "No you haven't. The sink's not wet." And, "well I did it. I did do it, I did it when I got to my…' Because he's a sink in his room, "I did it when I got to my room" which we know is not necessarily the case".* (int 1) *"I tried to implement the isolation of amazon parcels and mocked by my teenage daughter!"* (s46)
**Habit forming reduces effort**		Descriptions of behaviours becoming easier when highly practiced/ingrained	*"Anything I can wipe down, I wipe down. So that, now… it, I mean, it is… it still is harder than it used to be, because I never would've done that before. But it is more normal now".* (int 3) *"Good advice needs to be followed but sometimes we forget. It needs to be made into a habit."* (s78)
**Confidence in how to perform the behaviours**		Inconsistent and confusing information undermines confidence. Concise information	*"I've just read an article in the Times that this [washing fresh produce in soapy water] is very dangerous, so I'll have to re-read it and decide, or*

For peer review only

		and validation increases confidence in how to perform the behaviours	*maybe you can tell me, because I've got no idea now, I'm completely confused".* (int 5)<br><br>"*So it's more if that, that's… it's attractiveness of thinking, 'yes, I… you know, if that's the kind of thing I'm doing and, you know, health experts are saying yeah, that's right...*" (int 2)<br><br>*"The advice helps to empower you, that we are not completely defenseless against deadly germs, we can be pro- active in stopping these germs making us ill.."* (s110)
**Social norms affect motivation to engage in the behaviours**		Includes any descriptions about others' perceived behaviour and its' effect on people's willingness to perform effortful behaviours	*"It starts being more relatable, and you can start imagining yourself putting those changes into practice. Like when the guy said his wife is always going round with the anti-bac, you can sort of imagine yourself doing that."* (int 12)

For peer review only

Standards for Reporting Qualitative Research (SRQR)*

http://www.equator-network.org/reporting-guidelines/srqr/

Title and abstract

	Page/line no(s).
**Title** - Concise description of the nature and topic of the study Identifying the study as qualitative or indicating the approach (e.g., ethnography, grounded theory) or data collection methods (e.g., interview, focus group) is recommended	p.1
**Abstract** - Summary of key elements of the study using the abstract format of the intended publication; typically includes background, purpose, methods, results, and conclusions	p.2

Introduction

**Problem formulation** - Description and significance of the problem/phenomenon studied; review of relevant theory and empirical work; problem statement	p.3-4
**Purpose or research question** - Purpose of the study and specific objectives or questions	p.4

Methods

**Qualitative approach and research paradigm** - Qualitative approach (e.g., ethnography, grounded theory, case study, phenomenology, narrative research) and guiding theory if appropriate; identifying the research paradigm (e.g., postpositivist, constructivist/ interpretivist) is also recommended; rationale**	p.9
**Researcher characteristics and reflexivity** - Researchers' characteristics that may influence the research, including personal attributes, qualifications/experience, relationship with participants, assumptions, and/or presuppositions; potential or actual interaction between researchers' characteristics and the research questions, approach, methods, results, and/or transferability	p.7-8
**Context** - Setting/site and salient contextual factors; rationale**	p.5,6,9-8
**Sampling strategy** - How and why research participants, documents, or events were selected; criteria for deciding when no further sampling was necessary (e.g., sampling saturation); rationale**	p.4-6
**Ethical issues pertaining to human subjects** - Documentation of approval by an appropriate ethics review board and participant consent, or explanation for lack thereof; other confidentiality and data security issues	p.8
**Data collection methods** - Types of data collected; details of data collection procedures including (as appropriate) start and stop dates of data collection and analysis, iterative process, triangulation of sources/methods, and modification of procedures in response to evolving study findings; rationale**	p.7-10

For peer review only - http://bmjopen.bmj.com/site/about/guidelines.xhtml

**Data collection instruments and technologies** - Description of instruments (e.g., interview guides, questionnaires) and devices (e.g., audio recorders) used for data collection; if/how the instrument(s) changed over the course of the study	p.7-9
**Units of study** - Number and relevant characteristics of participants, documents, or events included in the study; level of participation (could be reported in results)	p.5,7-9
**Data processing** - Methods for processing data prior to and during analysis, including transcription, data entry, data management and security, verification of data integrity, data coding, and anonymization/de-identification of excerpts	p.8-10
**Data analysis** - Process by which inferences, themes, etc., were identified and developed, including the researchers involved in data analysis; usually references a specific paradigm or approach; rationale**	p.9-10
**Techniques to enhance trustworthiness** - Techniques to enhance trustworthiness and credibility of data analysis (e.g., member checking, audit trail, triangulation); rationale**	p.9.10

Results/findings

**Synthesis and interpretation** - Main findings (e.g., interpretations, inferences, and themes); might include development of a theory or model, or integration with prior research or theory	p.10-22
**Links to empirical data** - Evidence (e.g., quotes, field notes, text excerpts, photographs) to substantiate analytic findings	p.10-22

Discussion

**Integration with prior work, implications, transferability, and contribution(s) to the field -** Short summary of main findings; explanation of how findings and conclusions connect to, support, elaborate on, or challenge conclusions of earlier scholarship; discussion of scope of application/generalizability; identification of unique contribution(s) to scholarship in a discipline or field	p.22-25
**Limitations** - Trustworthiness and limitations of findings	p.24

Other

**Conflicts of interest** - Potential sources of influence or perceived influence on study conduct and conclusions; how these were managed	p.25
**Funding** - Sources of funding and other support; role of funders in data collection, interpretation, and reporting	p.25

*The authors created the SRQR by searching the literature to identify guidelines, reporting standards, and critical appraisal criteria for qualitative research; reviewing the reference lists of retrieved sources; and contacting experts to gain feedback. The SRQR aims to improve the transparency of all aspects of qualitative research by providing clear standards for reporting qualitative research.

For peer review only

**The rationale should briefly discuss the justification for choosing that theory, approach, method, or technique rather than other options available, the assumptions and limitations implicit in those choices, and how those choices influence study conclusions and transferability. As appropriate, the rationale for several items might be discussed together.

Reference:

O'Brien BC, Harris IB, Beckman TJ, Reed DA, Cook DA. **Standards for reporting qualitative research: a synthesis of recommendations.** *Academic Medicine*, Vol. 89, No. 9 / Sept 2014 DOI: 10.1097/ACM.0000000000000388

For peer review only

BMJ Open

Infection control in the home: A qualitative study exploring perceptions and experiences of adhering to protective behaviours in the home during the COVID-19 pandemic

Journal:	*BMJ Open*
Manuscript ID	bmjopen-2021-056161.R1
Article Type:	Original research
Date Submitted by the Author:	04-Nov-2021
Complete List of Authors:	Morton, Katherine; University of Southampton, School of Psychology<br>Towler, Lauren; University of Southampton, School of Psychology<br>Groot, Julia; University of Bath, Department of Psychology<br>Miller, Sascha; University of Southampton, School of Psychology<br>Ainsworth, Ben; University of Bath, Department of Psychology; University of Southampton, NIHR Biomedical Research Centre, Faculty of Medicine<br>Denison-Day, James; University of Southampton, School of Psychology<br>Rice, Cathy; University of Southampton<br>Bostock, Jennifer; Quality and Outcomes of Person-Centred Care Policy Research Unit<br>Willcox, Merlin; University of Southampton, Primary Care Research Centre<br>Little, Paul; University of Southampton, Primary Care Research Centre<br>Yardley, Lucy; University of Southampton, School of Psychology; University of Bristol, School of Psychological Science
**Primary Subject Heading**:	Infectious diseases
Secondary Subject Heading:	Public health, Qualitative research
Keywords:	COVID-19, Infection control < INFECTIOUS DISEASES, QUALITATIVE RESEARCH, Public health < INFECTIOUS DISEASES

SCHOLARONE™
Manuscripts

I, the Submitting Author has the right to grant and does grant on behalf of all authors of the Work (as defined in the below author licence), an exclusive licence and/or a non-exclusive licence for contributions from authors who are: i) UK Crown employees; ii) where BMJ has agreed a CC-BY licence shall apply, and/or iii) in accordance with the terms applicable for US Federal Government officers or employees acting as part of their official duties; on a worldwide, perpetual, irrevocable, royalty-free basis to BMJ Publishing Group Ltd ("BMJ") its licensees and where the relevant Journal is co-owned by BMJ to the co-owners of the Journal, to publish the Work in this journal and any other BMJ products and to exploit all rights, as set out in our licence.

The Submitting Author accepts and understands that any supply made under these terms is made by BMJ to the Submitting Author unless you are acting as an employee on behalf of your employer or a postgraduate student of an affiliated institution which is paying any applicable article publishing charge ("APC") for Open Access articles. Where the Submitting Author wishes to make the Work available on an Open Access basis (and intends to pay the relevant APC), the terms of reuse of such Open Access shall be governed by a Creative Commons licence – details of these licences and which Creative Commons licence will apply to this Work are set out in our licence referred to above.

Other than as permitted in any relevant BMJ Author's Self Archiving Policies, I confirm this Work has not been accepted for publication elsewhere, is not being considered for publication elsewhere and does not duplicate material already published. I confirm all authors consent to publication of this Work and authorise the granting of this licence.

For peer review only

Infection control in the home: A qualitative study exploring perceptions and experiences of adhering to protective behaviours in the home during the COVID-19 pandemic

Katherine Morton[1¶], Lauren Towler[1*¶], Julia Groot[2], Sascha Miller[1], Ben Ainsworth[2,3], James Denison-Day[1], Cathy Rice[1,4], Jennifer Bostock[5,6], Merlin Willcox[7], Paul Little[7], & Lucy Yardley[1,8]

[1]School of Psychology, University of Southampton, Southampton, United Kingdom

[2]Department of Psychology, University of Bath, Bath, United Kingdom

[3]National Institute for Health Research Biomedical Research Centre, Faculty of Medicine, University of Southampton, Southampton, United Kingdom

[4]Public Contributor, Bristol, United Kingdom

[5]Quality Safety & Outcomes Policy Research Unit, University of Kent & Oxford, Kent, United Kingdom

[6]Public contributor, London, United Kingdom

[7]Primary Care Research Centre, University of Southampton, Southampton, United Kingdom

[8]School of Psychological Science, University of Bristol, Bristol, United Kingdom

* Corresponding author

Postal address: B44 University Road, Southampton, SO17 1BJ

Email: lbt1g14@soton.ac.uk

¶ KM and LT are joint first authors.

Word count: 6051

For peer review only

Abstract

Objectives: We sought to explore people's experiences and perceptions of implementing infection control behaviours in the home during the COVID-19 pandemic, guided by an online behavioural intervention.

Design: Inductive qualitative study

Setting: UK public during the COVID-19 pandemic

Participants: Thirteen people took part in telephone interviews, and 124 completed a qualitative open-text survey. All were recruited from the public. Most survey participants were aged over 60, while interview participants were more distributed in age. Most reported being at increased risk from COVID-19, and White British.

Intervention: Online behavioural intervention to support infection control behaviours in the home during the COVID-19 pandemic.

Data collection: Telephone think-aloud interviews and qualitative survey data.

Data analysis: The think-aloud interview data and qualitative survey data were analysed independently using inductive thematic analysis. The findings were subsequently triangulated.

Results: Thematic analysis of the telephone interviews generated 7 themes*: perceived risk; belief in the effectiveness of protective behaviours; acceptability of distancing and isolation; having capacity to perform the behaviours; habit forming reduces effort; having the confidence to perform the behaviours; and social norms affect motivation to engage in the behaviours.* The themes identified from the survey data mapped well onto the interview analysis. Isolating and social distancing at home were less acceptable than cleaning and handwashing, influenced by the need for intimacy with household members. This was

For peer review only

especially true in the absence of symptoms and when perceived risk was low. People felt more empowered when they understood that even small changes, such as spending *some* time apart, were worthwhile to reduce exposure and lessen viral load.

Conclusions: The current study provided valuable insight into the acceptability and feasibility of protective behaviours, and how public health guidance could be incorporated into a behaviour change intervention for the public during a pandemic.

Keywords: COVID-19, Infection control, Perceptions, Attitudes, Health Behaviours, Qualitative

STRENGTHS AND LIMITATIONS OF THIS STUDY

- To our knowledge, this is the first paper to qualitatively explore attitudes toward and experiences of performing protective behaviours within the home to prevent within-household transmission, which has been shown to be a key risk.
- Think-aloud interview data were triangulated with data from 124 qualitative survey respondents, and affinity between the two data sources was high.
- Transferability of the results is potentially limited due to the rapidly shifting nature of the pandemic, and limited representation of participants from minority ethnic groups.
- In addition, the qualitative survey had a low response rate which could limit transferability.

INTRODUCTION

Behavioural measures have been recommended to help control the spread of the COVID-19 virus, including hand-washing, cleaning surfaces, mask-wearing, social isolation, and social distancing.[1]). However, evidence suggests that adherence to these behaviours varies widely in the UK and other affected countries, suggesting there may be challenges for people in

For peer review only

implementing these behaviours in a real life setting.[2-4] Transmission of COVID-19 within the home is a key risk,[5,6] therefore understanding barriers to adhering to protective behaviours within the home could be particularly important.

Germ Defence is an infection control intervention which was initially developed using theoretical modelling and qualitative research to target seasonal colds and flu, in line with the person-based approach.[7] The intervention has been updated and optimised by the Universities of Bristol, Bath and Southampton to help people protect themselves at home from COVID-19,[2,8] and its implementation into primary care is currently being trialled.[9] During the development of Germ Defence, the theory of planned behaviour (TPB) was applied to identify behavioural determinants on which to base the content.[10] Leventhal's common-sense model of health and illness was used to ensure the website content attended to common perceptions and constructions of illness and infection.[11] To increase users' perceived risk, the intervention is structured using protection motivation theory (PMT) by emphasising the personal and social health consequences of contracting COVID-19.[12] Evidence suggests that TPB and PMT concepts in particular explain behavioural responses during a pandemic.[13] Risk messages are followed by supportive coping messages explaining how users can reduce that risk by lowering their contact with the virus. The language used on the website is in line with self-determination theory to increase users' motivation to carry out the behaviours.[14] Intervention content, design and structure was informed by qualitative think-aloud interviews with the general public.[15]

This study sought to explore experiences and perceptions of performing protective behaviours at home in order to identify possible barriers and facilitators, and develop an understanding of how these behaviours are influenced by perceptions. This forms part of the person-based approach to adaptation and optimisation of the Germ Defence intervention for COVID-19.[16]

For peer review only

METHOD

Participants

Inclusion criteria were those over the age of 18, able to access the Germ Defence website and able to give informed consent. Users of the Germ Defence website were invited to register their interest in taking part in research to optimise the website.

Interviews

Seven interview participants were purposively sampled from the volunteers by factors such as age, gender, education level, risk status and experience of COVID-19 to maximise diversity.

However, after seven interviews we identified that these participants were mostly highly educated about infection control behaviours and highly motivated to adhere. As we wanted to understand barriers amongst people with lower levels of awareness and motivation, we recruited the remaining participants via social media and newsletters sent out by organisations and community groups to target people who had not already sought out the Germ Defence intervention (n=6). We stopped recruiting once we felt we had reached saturation and that no new barriers or facilitators were being identified.

Survey

Users of Germ Defence who volunteered to participate in research but were not purposively sampled for an interview were invited to complete a short survey instead.

Measures

Demographics

Potential participants were asked to complete an online survey to determine age, gender, experience of COVID-19, education, household size, postcode to inform Index of Multiple

For peer review only

Deprivation, and ethnicity. Finally, contact information was collected to enable a researcher to invite the potential participant to interview or to complete the survey.

Interview topic Guide

Think-aloud semi-structured interviews[17,18] were conducted by three female interviewers (LT, KM and JG), in which the participants provided feedback on each page of the online intervention (https://www.germdefence.org/) to provide detailed insights into their perceptions of the content.[2] At the beginning of the interviews, participants were asked a series of questions pertaining to their general perceptions of COVID-19 and protecting themselves at home (e.g "Can you tell me how you feel about the coronavirus at the moment?"). Then, the participants used the website and the researcher asked them what they thought of the content on each page. All interviewers were researchers within the field of health psychology. Prompts or follow-up questions typically pertained to attitudes toward the behavioural information and determinants of engagement and adherence. At the close of the interview, a series of general questions were asked about their overall views of the Germ Defence website.

Survey

The qualitative survey featured four open-text questions in addition to closed demographics questions. The survey aimed to gather participants' thoughts on the protective behaviours suggested on the website such as, "How do you feel about following the suggestions on Germ Defence?" and "What did you not like about the Germ Defence advice?"

Procedure

Those who accessed the Germ Defence website and completed at least one section saw a pop-up banner asking if they might be interested in taking part in research to help improve the website. If they indicated they wished to take part in research they were asked to complete

For peer review only

the online demographic questions hosted by Qualtrics to inform purposive sampling. In addition, adverts inviting people to take part in a telephone interview about a website designed to help keep them and their household safe from coronavirus were posted on social media, with a link to the purposive sampling questions. Ethical approval was granted by the University of Southampton Psychology Ethics Committee (ID: 56445).

Interviews

Participants were purposively selected by the research team and sent a link to the information sheet and consent form, which was completed online. Interviews were conducted by telephone, due to the pandemic. The audio recording began once consent was verbally reaffirmed. At the close of the interview, participants were thanked with an Amazon voucher. The interviews took place during a period of rapidly changing guidelines in the UK, from 8th June to 5th November 2020, most whilst the R-rate was relatively low, and restrictions were soon to be (or had already been) lifted.

Survey

A total of 545 respondents were invited to complete the survey over three separate mail-outs: the first on 19th June 2020 (n=150); the second on 10th July 2020 (n=103); and the third on 24th July 2020 (n=292). The email contained a link to the survey, which began with a participant information sheet and consent form. For context, the first mail-out occurred during the first lockdown, which was lifted on 4th July 2020, but wearing face-coverings inside shops only became compulsory on the date of the final mail-out; 24th July 2020.

Patient and Public Involvement (PPI)

As Germ Defence is available to the general public, PPI was integral to its development. Two public contributors (CR and JB) on our stakeholder panel participated in weekly meetings which informed the optimisation of the intervention, and worked with us to identify potential

For peer review only

issues in the behavioural messages of the intervention and update the intervention content in line with feedback. The conceptualisation, measures, recruitment strategy and dissemination of the current study was informed by open discussion with these members. For example, the public contributors reviewed the interview topic guide and assisted in identifying which organisations to target during the recruitment process. In particular, the public contributors provided considerable assistance in ensuring that the study materials and study invitations were easy to understand and free of jargon. Further detail on PPI in the development and optimisation of Germ Defence has been reported elsewhere.[16]

Data Analysis

Interviews

Data were analysed using inductive thematic analysis to openly explore the barriers and facilitators that were important to people.[19,20] Due to the need for rapid analysis and dissemination of initial findings, the first set of transcripts were split between two researchers (n = 6 transcripts analysed by KM and n = 3 transcripts by LT). The researchers independently read their transcripts thoroughly to first familiarise themselves with the data. Data were then coded inductively by unit of meaning using NVivo, keeping the core aims of the study in mind (barriers and facilitators to, and perceptions of, infection control behaviours in the home). After the first nine interviews had been coded, the researchers met and compared their coding manuals, discussing each code and theme in detail and generating a final agreed coding manual to unite their coding. This involved revisiting the raw data to confirm shared and consistent understanding of how the codes and themes were being used. The coding manual was then used by LT to code the remaining four interviews, and where necessary new codes were added and existing codes were further refined, although these amendments were only minor. LT double-checked the earlier transcripts to ensure the revised

For peer review only

coding manual was consistently applied across the data, and the researchers met again to confirm agreement on the final coding manual. Findings were shared with participants via a newsletter, and participants were invited to contact the research team if they had any feedback on the findings.

Survey

Responses to the four open-text survey questions were coded inductively using thematic analysis, separately from the interview data analysis. The resulting categories were then mapped onto the themes generated from the interview data to assess their fit with these themes, whether any new themes or subthemes were present in the survey data, and to what extent the survey data provided further nuance to the existing themes. Inductive coding was deemed most appropriate, as the researchers intended to triangulate the two datasets for complementarity, rather than convergence, to ensure that any unique perspectives gathered from the survey data were attended to.

RESULTS

Interviews

Table 1 shows the demographic details of the 13 interview participants. The mean interview length was 79 minutes (range 60-104 minutes). Most participants lived with at least one other person, and 7 participants felt that either they or a household member was at increased risk should they contract the virus.

Table 1. Interviewee demographics.

ID	Sex	Age	Date interviewed	Household members
1	F	61-70	08/06/2020	Lives with spouse and teenage children
2	F	61-70	11/06/2020	Lives with husband with cancer

For peer review only - http://bmjopen.bmj.com/site/about/guidelines.xhtml

3	F	41-60	12/06/2020	Lives with teenage children
4	F	61-70	29/06/2020	Lives alone
5	F	41-60	01/07/2020	Lives with older parents with comorbidities, spouse, and teenage child
6	F	61-70	03/07/2020	Lives with partner
7	F	41-60	07/07/2020	Lives with spouse and adult son
8	F	41-60	16/07/2020	Lives alone
9	M	18-25	23/07/2020	Lives with parents and sister
10	M	26-40	10/09/2020	Lives with partner
11	F	61-70	21/09/2020	Lives with husband with comorbidities
12	F	26-40	28/09/2020	Lives with partner
13	F	26-40	05/11/2020	Lives with partner

Survey

A total of 124 website users completed the qualitative survey (n = 545 invited, 23% response rate). Most participants were over 60 years old, reported being at increased risk from COVID-19, and White British. Table 2 shows the demographic details of the survey respondents.

For peer review only

Table 2. Survey respondent demographics

		N	%
Age	26-40	2	1.6
	41-60	37	29.8
	61-70	41	33.1
	70+	31	25
	Missing	13	10.5
Experience with COVID-19			
	I am at increased risk	50	40.3
	Someone I live with is at increased risk	19	15.3
	I think I've had COVID-19	7	5.6
	I think someone I live with has had COVID-19	1	0.8
	None of the above/No experience	33	26.6
	Unassigned	14	11.3
Ethnicity	White British	101	81.5
	White Irish	1	0.8
	White European	2	1.6
	White Canadian	2	1.6
	Black British	1	0.8

For peer review only

	Black African	1	0.8
	British Chinese	1	0.8
	Missing	15	12.1
Education level	Pre-secondary school	1	0.8
	Secondary School	43	34.7
	Undergraduate	38	30.6
	Postgraduate	28	22.6
	Missing	4	11.3

The researchers generated 7 key themes from the interview data related to perceived barriers and facilitators to engaging with infection control behaviours in the home. These were: *perceived risk*; *belief in the effectiveness of protective behaviours*; *acceptability of distancing and isolation*; *having capacity to perform the behaviours*; *habit forming reduces effort*; *confidence in how to perform the behaviours*; and *social norms affect motivation to engage in the behaviours*. See Supplementary File 1 for the coding manual. Extracts from the interview data are delineated by the abbreviation 'int'.

For the qualitative survey, most respondents felt positively about the protective behaviours recommended on the Germ Defence website. The themes identified from the survey data mapped well onto the interview analysis, with particularly strong congruence to *confidence in how to perform the behaviours.* The survey findings are discussed alongside the interview data within the themes which they mapped onto. Extracts from the survey data are delineated by the letter 's'.

Perceived risk

For peer review only

Germ Defence encourages users to evaluate their own level of risk and which actions they feel are appropriate for them based on this level of risk, to enable users to focus on the behaviours and advice they deem the most personally relevant. For more detail on the intervention content and how we tailored it for perceived risk, see other publications from the project.[2,21] Participants' assessments of their level of risk played a major role in their willingness to engage in the protective behaviours, particularly those seen as more 'extreme' such as social distancing from other household members. Those who perceived that the virus is likely to enter their home, and/or that household members are at risk of becoming seriously unwell were generally highly motivated to engage with the behaviours.

Current levels of virus in circulation

Information about the current actual risk of infection was important for some people to help make decisions about performing difficult behaviours. For example, a mother justified her reluctance to follow social distancing guidance in the home in terms of the lower perceived necessity to do this at the moment.

> *"There is that sort of hope that, as there is I think known to be that much less of the virus out there generally at the moment… although we're still taking all the precautions, there is that hopefulness that the risk is less now than it was back in March."* (int 3)

> *"I didn't follow the stricter suggestions such as using disinfectant in the home, as we're low risk and the area we live in has very low numbers of cases.*" (s71)

Perceived likelihood of virus entering the home

Some participants were concerned about those in the household bringing the virus home if they needed to leave for work. This was influenced by how much mixing the person was

For peer review only

doing outside the home, and the perceived severity of the consequences if someone in the household became ill.

> *"They said only one person is allowed out during the lockdown. So it was my husband… I was worried, because I'm the one who does the cooking and things, that I would pass it on to my parents if he caught it."* (int 5)

Having people from outside the household in the home was felt to be a significant risk. Participants were generally highly motivated to engage in the protective behaviours when visitors were present.

> "*I had a workman come in and he had to look at – because my heating's gone – and I was having a heart attack with him touching anything. So I was going round spraying everything with bleach like a maniac, even the carpet. So what are you meant to do if you've got workmen. I made him wear a mask, I made him wear gloves.*" (int 5)

> "*Well I'm not going in anybody's house, and I'm not having anybody in my house…My house is my safe haven."* (int 4)

Perceived risk of severe consequences to health

People's perceived risk of severe illness or death from the virus was influenced by co-morbidities (such as cancer, COPD, asthma, and high blood pressure), old age, ethnicity, and being an inter-generational household.

> *"When you've taken a decision to tell your parents to come and live with you, and then you're reading stuff about intergenerational households, it's a much higher risk…"* (int 5)

One participant described how she decided to shield with her husband to protect him, despite not being classed as vulnerable herself.

For peer review only

"I would just be so petrified I was going to give him something… I feel less… kind of imprisoned in a way, by shielding myself with him, than going out into the so-called freedom, but then coming back and being petrified I'll kill him." (int 2)

Another described how one of the younger members of her household felt he didn't need to worry about the virus because of his age, and he perceived that only those at increased risk needed to be concerned.

"Our young man thinks that the only people that you should be worried about are people that are at increased risk, should they catch it. Not everybody else. Do you know what I mean, it's like, oh well, it doesn't matter because they're fine, my friends are fine." (int 1)

Belief in the effectiveness of the protective behaviours

The perceived effectiveness of behaviours appeared to influence participants' willingness to engage with them. Participants identified an important caveat: the virus could spread within the home before symptoms present, meaning that protective behaviours could be viewed as pointless unless performed consistently. However, perceiving viral load to be a factor in viral transmission seemed to mitigate this, and these participants felt empowered to enact small changes around their home to reduce their risk.

Perceived value of cleaning

Most participants were already very aware of cleaning and washing hands and felt these were important. However, cleaning was sometimes associated with being paranoid and fearful, and some participants were keen to explain they weren't paranoid about the level of cleaning they do, whilst others described how the virus has made them feel paranoid about cleaning.

For peer review only

"Careful but not paranoid, yeah. I don't wash my keys in soapy water, and I don't regularly wash my car. We just wash and hand gel our hands after we've been somewhere that's in the car, when we get back into it." (int 7)

"at the beginning I was cleaning constantly. I still am…. And then I'm spraying down the surfaces with disinfectant, because I'm worried about this transference. Okay, you've just touched it, so you've put it down. So that now gets onto that surface, if somebody in the meantime touches that surface, it then carries on and then goes onto another surface. That's what I'm on about, with the paranoia." (int 5)

Perceived value of wearing a face-covering

People's willingness to wear a face-covering was strongly influenced by perceptions of effectiveness, although the focus was on wearing them outside the home. Most of the interviews took place prior to the mandatory use of face-coverings in the UK, and there was some uncertainty and variance within the public discourse regarding their effectiveness at the time. These sentiments were reflected by our participants. Some people had read information from other countries which convinced them that face-coverings were an effective way to prevent transmission, and one participant emphasised how she believed face-coverings were important for protecting others more than yourself, whereas a few remained unconvinced and wanted more evidence.

"*I might wear a mask, like I told you, I need to do more research on that.*" (int 6)

Reasons offered for why masks might be ineffective included lack of filters, the mask causing infection due to dampness from breath, and people touching their face. Furthermore, at the start of the pandemic and during the time in which most of the data collection took place, infection control strategies (including Germ Defence) placed a strong focus on surface transmission. As the pandemic progressed, the focus has shifted to airborne transmission,

For peer review only

particularly the importance of ventilation. However, since manual transmission remains a potential transmission pathway within the home, Germ Defence was altered to additionally emphasise airborne transmission, rather than reduce the emphasis on handwashing and surface transmission. For more information on the advice given in Germ Defence and how this has changed during the progression of the pandemic based on PHE, PPI and stakeholder input, see further publications from the project.[16,21] This could explain why our participants reported stronger beliefs in the value of cleaning surfaces over face-covering and ventilation.

Barrier: Virus is likely to spread before you know you're ill

Some people were uncertain whether it would be achievable to prevent the virus spreading in the home.

> *"I think I probably still am, to a certain extent, sceptical about whether we would be able to get a virus come into this home and avoid spreading it between us."* (int 3)

People were concerned that the virus would already have spread by the time they socially distanced or self-isolated, making it pointless unless done continually.

> *"If at any stage I started to feel ill, which is probably then too late, because I probably would've then spread it to them, I could've potentially spread it to them by then anyway, I would then take myself to my room."* (int 3)

Facilitator: Reducing all or nothing thinking

People were more likely to perceive protective behaviours as effective and worthwhile when they perceived catching the virus as a continuum based on how much viral load you are exposed to, rather than you either catch it or not.

For peer review only

> *"I use antibacterial wipes on just about all the shopping that comes into the house as well, when it's delivered, just as a precaution. Because I think it's safer if you do get the virus that it's as small as possible."* (int 7)

This was empowering as it helped people feel that small changes can still make a difference.

> *"I am sitting here thinking, if I turned the table the other way around, we could actually sit further apart from each other at the table, which might be one small thing."* (int 3)

Survey participants also highlighted the importance of balancing behaviours in accordance with personal risk level and perceived negative impact of the behaviour (e.g. social distancing negatively impacting wellbeing), linking in with the perceived risk theme.

> "*It might not be good to be keeping them* [children] *at 2 m away for their development or mental health. Need more nuances about balancing risk against looking after child development."* (s18)

Acceptability of distancing and isolation

Social distancing and isolation behaviours were presented on the Germ Defence website as recommended for higher risk individuals, but also as useful ideas for lower risk households to help reduce risk whenever it was deemed necessary. Spending time together was perceived as integral to the wellbeing of the household, but some participants described small changes they had made to help maintain intimacy while social distancing or self-isolating. Social distancing and self-isolation were seen by some as only acceptable for short periods of time when symptoms were present.

Barrier: Importance of time together

For peer review only

The idea of self-isolating within the home was quite daunting for people and there was some concern about the effect upon mental wellbeing. Experiences of intimacy with partners and family members was generally judged to be of higher importance than reducing the risk of virus transmission when no symptoms were present, even when some members of the household were high risk.

> *"I don't think I could cut down on the amount of time I spend with other people, because they'll get lonely…"* (int 5)

> *"Because to a ninety-five-year-old a kiss is more important than worrying about whether or not you're going to die of a virus."* (int 11)

Some people described spending some time on their own during the day, but the evening meal was often regarded as an important time to spend together.

> *"The evening meals are nice… that's the one thing where we don't really take any precaution with the family, just because we all sit around the dinner table. But that is a nice part of the day, really, so in that respect it's quite good for everyone's mental health."* (int 9)

One couple found the idea of eating separately with the at-risk individual in his room as completely unacceptable:

> *"I think the guidance said something awful, like he should stay in his own room and be, you know, deliver his food to him like he was a kind of caged animal."* (int 2)

Some people perceived social distancing as acceptable for short periods of time if someone is ill, but not as something to do indefinitely as a preventative measure.

> *"Is that something I would have to do all the time, every day of my life? And then that feels completely… I wouldn't feel that there was much quality of life if I had to… if*

For peer review only

I'm living in the same house as my children at the moment but I couldn't hug them or sit near them or… It's something I could see potentially doing if it was for a limited period, but it just feels impossible sort of long-term." (int 3)

Facilitator: Ways of maintaining (distanced) intimacy

Some participants had made changes at home to enable social distancing, and they described how they managed to maintain some feelings of intimacy. Small changes to furniture arrangements or daily routines, the use of technology, and contact which was perceived to be low risk were seen as effective ways to engage with the protective behaviours without completely sacrificing intimacy and connectedness:

"I added on an extra table in the dining room, so that I could keep a metre from him when we're eating, even though it's joined eating." (int 5)

"We have a bit of a dry cuddle, like I go over his shoulders, but I don't breathe on him and he doesn't breathe on me. So we're kind of on board with it, you know?" (int 2)

"In the morning, I go and wake him up and say, "Oh, I'm getting up now for work," and he goes down and makes me a cup of tea, just because we kind of like to have that... But he will deliver it to my dressing table and then I'll pick it up and take it back to bed. It's kind of trying to keep that intimacy, but without actually sharing everything." (int 2)

Having capacity to perform protective behaviours

This theme explores participants' perceptions of the practical factors which affect their capacity to perform the suggested behaviours. Having sufficient space was an important

For peer review only

factor in how feasible it was for people to socially distance and self-isolate. Those who lived in smaller spaces generally found the idea of social distancing unfeasible.

> "*I'm guessing this applies to people in like houses more than just like one room, 'cause I currently live in a flat, a one bed flat with my partner, so it's kind of impossible for us to have one room in our home to be just for us.*" (int 12)

Some people found it challenging trying to implement house rules for others to follow during the pandemic. Handwashing was a particular behaviour mentioned that participants tried to persuade partners and children to do, or checked whether they had done, which was identified as a source of tension.

> *"But when he comes home, I tell him to wash his hands, and every time he gets home, I'm always, "Have you washed your hands?"* (int 6)

> *"I will just keep reminding him, all the time, to wash his hands. And he'll say, "I've done it." You say, "No you haven't. The sink's not wet." And, "well I did it. I did do it, I did it when I got to my...' Because he's a sink in his room, "I did it when I got to my room" which we know is not necessarily the case. So it's… it's tricky, but we're trying to keep on the case."* (int 1)

Habit forming reduces effort

When discussing the effort involved in performing the protective behaviours, participants typically discussed how well they integrated with their current behaviours and routines. Some participants described how some protective behaviours, such as cleaning, regular handwashing and not sharing towels, had already been the norm for them before the pandemic, which helped them to adhere.

For peer review only

"I found that they are things that I have always done, throughout my life, because I was taught to as a child." (int 4)

Social distancing was also facilitated in some households with teenage children, who were described as spending a lot of time in their rooms anyway. Additionally, despite an initial negative reaction to social distancing, some described how working from home meant that they were spending most of their time away from other household members.

"*I'm looking at it going, 'Really? You think this is a rational thing to do?' Like I mean, I do sit in a room on my own for most of the day, funnily, because I work from home, as does my partner, and you know, it… she'd irritate me if she was on calls and vice versa, so yeah, we do sit separately.*" (int 10)

Where new behaviours had become habits for our participants, they perceived less effort involved in performing the behaviours.

"I think they're definitely becoming habits now. I mean, it is… still is harder than it used to be, because I never would've done that before. But it is more normal now." (int 3)

Others who were being extremely careful about cleaning found it could be quite effortful and fatiguing. It seemed that participants living with people at increased risk were more likely to find the constant cleaning demanding.

"It feels like it's a constant state of vigilance. It's very high intensity, that level of concentration all the time, not to lapse." (int 5)

Confidence in how to perform the behaviours

For peer review only

Both interview and survey participants identified that they wanted clear and consistent practical information on what to do. Inconsistent information seemed to undermine people's confidence in their ability to perform the behaviours and reduce their risk.

> "*It's easier now than when it first started…I feel like the mask guidance just came out of nowhere, so one minute they're telling us that they don't have any scientific evidence, and the next minute it's, 'from the 30th you have to wear masks,'… it was just strange…*" (int 12)

However, both interview and survey participants felt more confident in their ability to engage with and perform the behaviours when they felt well informed and affirmed by those who they perceived to be experts.

> "*Knowing the advice came from trusted source gave me confidence and so helped to avoid fear/anxiety overwhelming.*" (s81)

When participants felt that they were doing the 'right' thing, they felt empowered and motivated to continue. The Germ Defence website encourages users to plan how much they intend to engage with the behaviours going forward. If their plans show that their adherence will improve, they are given positive reinforcement:

> "*It's quite validating…I've reconsidered what I've been doing and now I'm going to make the steps, and I feel quite empowered.*" (int 12)

Social norms affect motivation to engage in the behaviours

Some participants discussed how they felt demotivated to engage in the behaviours when they perceived others were disregarding infection control advice. These participants felt that protective behaviours were simply "not worth the effort" when others were not playing their part.

For peer review only

"I feel a little bit disenchanted by the whole thing, because you know, I've done things properly, … I didn't leave the house for… three months. And even when it was relaxed I didn't, and yet I still have to watch my neighbour, who's seventy-five, going out for a drive every single day during lockdown, and that is difficult to take. So it was a bit like 'actually … why am I doing my bit here, when everyone else isn't?'" (int 10)

DISCUSSION

These findings show how people conceptualise the risk of catching and transmitting COVID-19, and use this as a rationale for their behaviour at home. In line with Protection Motivation Theory[12] and a previous review of beliefs influencing protective behaviours during the swine flu pandemic,[13] perceived risk of the virus and perceived effectiveness of the protective behaviours increased willingness to adhere . Cleaning and handwashing were widely perceived to be effective and acceptable, although some participants described how other members of their household were less adherent to these behaviours which could cause anxiety. Participants also found the protective behaviours easier if they fit well with their usual routine, suggesting that linking the new behaviours to more ingrained habits could increase adherence.

Behaviours such as spending time in separate rooms at home and keeping two metres apart were less acceptable, especially as preventative measures to follow even when no-one in the household has any symptoms (although this was only suggested for higher risk households). Our participants generally felt that a lack of physical and emotional closeness with their household members was too much of a sacrifice to engage in social distancing regularly, even when the household was identified as high risk. Additionally, since our participants tended to find the behaviours easiest to adhere to when they fit well with their usual routine and when

For peer review only

they formed a habit, it could be that these particular behaviours are seen as too different from their typical way of life. Finally, awareness of the concept of viral load helped people feel more empowered as they understood that even small changes, such as spending *some* time apart, were worthwhile. This finding is consistent with the importance of *attitudes* and *perceived behavioural control* from the Theory of Planned Behaviour.

There was some congruency between the current findings and previous research into adherence to infection control behaviours during a pandemic. The concern about being perceived as paranoid (within the subtheme; *perceived value of cleaning)*, indicated that there may still be negative social connotations surrounding hygiene practices.[15] and supports the relevant of *social norms* from the TPB.[10] Further, our findings regarding the need for emotional connection and intimacy provide support for recent qualitative research into the impact of COVID-19 and adherence to government guidance, which showed that some may only partially adhere to the behaviours due to the need for and cultural importance of social contact, and some reported feelings of loss and grief over the loss of social interaction during lockdown.[22-24] Concerns about the negative impact of self-isolation, both in terms of practical logistics and emotional well-being, were also raised in a qualitative study with people who had been in contact with someone with COVID-19.[25] This suggests that self-isolation is a very difficult behaviour for many people even when risk is known to be high, and that appropriate support is essential. Additionally, the need for clarity and consistency in government and public health guidance has also been highlighted in other studies as important in aiding the public to adhere to infection control behaviours.[22,24,26]

Finally, our participants expressed some concern and awareness that transmission to other household members may well have occurred by the time that symptoms present, supporting previous qualitative research into the public's opinions of the need for separate accommodation for at-risk individuals during the COVID-19 pandemic.[27] This indicates a

For peer review only

need for preventative educational interventions so that the public are equipped to act as soon as they feasibly can.

Strengths and Limitations

Triangulation of the think-aloud data with open-ended survey data revealed very high affinity between the two datasets, suggesting that the themes identified are valid and robust. However, the transferability of our results should still be treated with some caution due to the rapidly changing nature of the pandemic and government guidance, and because our sample may not represent the views of the general population. Half of our interview participants were Germ Defence users, recruited after receiving the intervention. It is therefore likely that they were more engaged and motivated than the general population since they sought out the intervention for themselves and subsequently volunteered to participate in research. The interview data gathered from non-website users did not differ substantially from the website users' experiences, although these volunteers are also likely to have an above average interest in reducing transmission. Similarly, our survey had a low response rate of only 23%, suggesting that the findings may not be representative of the barriers to protective behaviours experienced by the wider population.

Our survey sample was also predominantly White Caucasian, and no interview participants identified themselves as belonging to Black, Asian and minority ethnic (BAME) groups. While efforts were made to purposively sample for greater diversity, the need for rapid data collection to inform the optimisation of the intervention limited our recruitment options. However, as noted above, some similar concepts to the current findings were found in a recent interview study which focused on members of low income and ethnic minority households.[23]

For peer review only

Our qualitative interviews were conducted via telephone due to the pandemic, but this remote method of data collection did not seem to negatively influence the richness or quality of the data. Participants appeared happy to share in-depth stories about their experiences and perceptions of the behaviours, and this is consistent with other research which has supported the value of remote qualitative research.[28]

Conclusions and Implications

Our findings have several implications for behavioural interventions and public health guidelines during a pandemic. These findings have shown that the public may be unwilling to adhere to the protective behaviours indefinitely if they perceive the risk to be low, so it is important that behavioural guidelines encourage accurate perceptions of personal risk level and highlight that enacting even small changes would still be worthwhile for reducing risk. People understood the concept of viral load and found this a helpful rationale for making small changes which could be maintained over time. Furthermore, the perceived negative impact of social distancing and isolation on mental wellbeing within the home seems to be a major sticking point in terms of the public's willingness to adhere. Behavioural interventions which offer practical suggestions for how intimacy could be maintained whilst socially distancing could reassure the public that they could reduce the negative impact on their wellbeing whilst engaging with protective behaviours, at least some of the time.

FUNDING STATEMENT

The study was funded by United Kingdom Research and Innovation Medical Research Council (UKRI MRC, https://mrc.ukri.org/) Rapid Response Call: UKRI CV220-009. The Germ Defence intervention was hosted by the Lifeguide Team, supported by the NIHR Biomedical Research Centre, University of Southampton. LY is a National Institute for Health Research (NIHR) Senior Investigator and theme lead for University of Southampton

For peer review only - http://bmjopen.bmj.com/site/about/guidelines.xhtml

Biomedical Research Centre. LY is affiliated to the National Institute for Health Research Health Protection Research Unit (NIHR HPRU) in Behavioural Science and Evaluation of Interventions at the University of Bristol in partnership with Public Health England (PHE). MW is an NIHR Academic Clinical Lecturer, under grant CL-2016-26-005.The views expressed are those of the author(s) and not necessarily those of the NHS, the NIHR, the Department of Health or Public Health England. The funders had no role in study design, data collection and analysis, decision to publish, or preparation of the manuscript.

COMPETING INTERESTS STATEMENT

The authors declare that no competing interests exist.

DATA SHARING STATEMENT

Data cannot be shared publicly because our participants did not consent to the publication of full transcripts. Only extracts are made available to those outside of the study research team. A coding manual containing additional extracts has been supplied as an online resource.

AUTHOR CONTRIBUTIONS

Katherine Morton

Conceptualization, Formal Analysis, Investigation, Methodology, Project Administration, Data Curation, Resources, Validation, Writing – Original Draft Preparation, Writing – Review & Editing

Lauren Towler

Conceptualization, Formal Analysis, Investigation, Methodology, Project Administration, Data Curation, Resources, Validation, Writing – Original Draft Preparation, Writing – Review & Editing

For peer review only

Julia Groot

Investigation, Project Administration, Writing – Review & Editing

Sascha Miller

Conceptualization, Resources, Writing – Review & Editing

Ben Ainsworth

Conceptualization, Funding Acquisition, Resources, Writing – Review & Editing

James Denison-Day

Software, Resources, Writing – Review & Editing

Cathy Rice

Resources, Writing – Review & Editing

Jennifer Bostock

Resources, Writing – Review & Editing

Merlin Willcox

Resources, Writing – Review & Editing

Paul Little

Conceptualization, Funding Acquisition, Resources, Writing – Review & Editing

Lucy Yardley

Conceptualization, Funding Acquisition, Resources, Writing – Review & Editing

ACKNOWLEDGEMENTS

For peer review only

We would like to thank our voluntary research assistants, Amina Khan and Lara Rosa, who led engagement with social media and charities for study recruitment.

ETHICS STATEMENT

This study involves human participants and was approved by the University of Southampton Ethics Committee (ID: 56445). Participants gave informed consent to participate in the study before taking part.

For peer review only

REFERENCES

1. West R, Michie S, Rubin GJ, et al. Applying principles of behaviour change to reduce SARS-CoV-2 transmission. Nat Hum Behav. 2020;4(5):451-9. doi: 10.1038/s41562-020-0887-9
2. Ainsworth B, Miller S, Denison-Day J, et al. Infection Control Behavior at Home During the COVID-19 Pandemic: Observational Study of a Web-Based Behavioral Intervention (Germ Defence). *J Med Internet Res*. 2021;23(2):e22197. doi: 10.2196/22197
3. Bodas M, Peleg K. Self-isolation compliance in the COVID-19 era influenced by compensation: Findings from a recent survey in Israel. *Health Aff*. 2020;39(6):936-41. doi: 10.1377/hlthaff.2020.00382
4. Machida M, Nakamura I, Saito R, et al. Adoption of personal protective measures by ordinary citizens during the COVID-19 outbreak in Japan. *Int J Infect Dis*. 2020;94:139-144. doi: 10.1016/j.ijid.2020.04.014
5. Wang Z, Ma W, Zheng X, et al. Household transmission of SARS-CoV-2. J Infect. 2020;81(1):179-82. doi: 10.1016/j.jinf.2020.03.040
6. Wang Y, Tian H, Zhang L, et al. Reduction of secondary transmission of SARS-CoV-2 in households by face mask use, disinfection and social distancing: a cohort study in Beijing, China. BMJ Glob Heal. 2020;5(5):e002794. doi:10.1136/bmjgh-2020-002794

7. Yardley L, Morrison L, Bradbury K, et al. The person-based approach to intervention development: Application to digital health-related behavior change interventions. *J Med Internet Res*. 2015;17(1):e30. doi: 10.2196/jmir.4055
8. Ainsworth B, Steele M, Stuart B, et al. Using an analysis of behavior change to inform effective digital intervention design: how did the PRIMIT website change hand hygiene behavior across 8993 users? *Ann Behav Med*. 2016;51(3):423-431. doi: 10.1007/s12160-016-9866-9
9. Horwood J, Chalder M, Ainsworth B, et al. Primary Care implementation of Germ Defence, a digital behaviour change intervention to improve household infection control during the COVID-19 pandemic: A structured summary of a study protocol for a randomised controlled trial. *Trials*. 2021;22(263). doi: 10.1186/s13063-021-05188-7
10. Ajzen I. The theory of planned behavior. *Organ Behav Hum Decis Process*. 1991;50:179–211. doi: 10.1016/0749-5978(91)90020-T
11. Leventhal HA, Brissette I, Leventhal EA. The common-sense model of self-regulation of health and illness. In: Cameron LD, Levanthal H, editors. The self-regulation of health and illness behaviour. London: Routledge 2003:42–65.
12. Rippetoe PA, Rogers RW. Effects of components of protection motivation theory on adaptive and maladaptive coping with a health threat. *J Pers Soc Psychol*. 1987;52(3). doi: 10.1037//0022-3514.52.3.596
13. Bish A, Michie S. Demographic and attitudinal determinants of protective behaviours during a pandemic: a review. *Br J Health Psychol*. 2010;15(4):797–824. doi: 10.1348/135910710X485826
14. Deci EL, Ryan RM. The "what" and the "why" of goal pursuit: Human needs and the self-determination of behavior. *Psychol Inq*. 1998;11:227–268. doi: 10.1207/S15327965PLI1104_01

For peer review only

15. Yardley L, Miller S, Teasdale E, et al. (2011). Using mixed methods to design a web-based behavioural intervention to reduce transmission of colds and flu. *J Health Psychol*. 2011;16(2):353–364. doi: 10.1177/1359105310377538

16. Morton K, Ainsworth B, Miller S, et al. Adapting behavioural interventions for a changing public health context: A worked example of implementing a digital intervention during a global pandemic using rapid optimisation methods. *Front Public Health*. 2021;9:668197. doi:10.3389/fpubh.2021.668197

17. Cotton D, Gresty K. Reflecting on the think-aloud method for evaluating e-learning. *Br J Educ Technol*. 2006;37(1):45-54. doi: 10.1111/j.1467-8535.2005.00521.x

18. Morrison L, Muller I, Yardley L, et al. The person-based approach to planning, optimising, evaluating and implementing behavioural health interventions. *Eur Heal Psychol*. 2018;20(3);464-469.

19. Braun V, Clarke V. Using thematic analysis in psychology. *Qual Res Psychol*. 2006;3(2):77-101. doi:10.1191/1478088706qp063oa

20. Clarke V, Braun V. Successful Qualitative Research: A Practical Guide for Beginners. London: Sage Publishing 2013.

21. Miller S, Denison-Day J. Applying the Person Based Approach for rapid optimisation of a digital intervention during a pandemic: adapting Germ Defence for COVID-19. Department of Psychology, University of Southampton. Forthcoming 2021.

22. Alipour F, Arshi M, Ahmadi S, et al. Psychosocial challenges and concerns of COVID-19: A qualitative study in Iran. *Health*. 2020;1363459320976752. doi: 10.1177/1363459320976752

23. Denford S, Morton KS, Lambert H, et al. Understanding patterns of adherence to COVID-19 mitigation measures: a qualitative interview study. *J Public Health*. 2021;fdab005. doi: 10.1093/pubmed/fdab005

For peer review only

24. Williams SN, Armitage CJ, Tampe T, et al. Public perceptions and experiences of social distancing and social isolation during the COVID-19 pandemic: a UK-based focus group study. *BMJ Open*. 2020;10(7):e039334. doi: 10.1136/bmjopen-2020-039334.

25. Denford S, Martin AF, Love N, et al. Engagement with daily testing instead of self-isolating in contacts of Confirmed cases of SARS-CoV-2: A qualitative analysis. *Front Public Health*. 2021;9:714041. doi: 10.3389/fpubh.2021.714041

26. Benham JL, Lang R, Kovacs Burns K, et al. Attitudes, current behaviours and barriers to public health measures that reduce COVID-19 transmission: A qualitative study to inform public health messaging. *PLOS ONE*. 2021;16(2):e0246941. doi: 10.1371/journal.pone.0246941

27. Denford S, Morton KS, Horwood J, et al. Preventing within household transmission of COVID-19: Is the provision of accommodation feasible and acceptable? *BMC Public Health*. 2021;21:1641. doi: 10.1186/s12889-021-11666-z

28. Drabble L, Trocki KF, Salcedo B, Walker PC, Korcha RA. Conducting qualitative interviews by telephone: Lessons learned from a study of alcohol use among sexual minority and heterosexual women. *Qual Soc Work*. 2016;15(1):118-133. doi:10.1177/1473325015585613

For peer review only

Supplementary File 1

Coding Manual

**Theme**	**Subthemes**	**Definition**	**Example quote**
**Perceived risk**	Current levels of virus in circulation	Weighing up perceived risk of virus based on current infection rates	*"I want to know is it safe for me to go out? You know, what's the transmission rate where I live?"* (int 6)
	Perceived likelihood of virus entering the home	Likelihood of a household member or someone outside the home bringing the virus into their home	*"the biggest concern is just bringing it in from outside, but I'm not doing particularly too much to risk that at the moment, I wouldn't say"*. (int 9)
	Perceived risk of severe consequences to health	Considering how vulnerable household members are to becoming severely ill from the virus	*"I'm fifty-three, going on fifty-four, and the age group is looking not so brilliant now, when I was looking in press reports. I know they said over seventy. I'm pretty fit, which is good. The only problem is, I have high blood pressure, and I'm on medication for that"*. (int 5)
**Belief in the effectiveness of the protective behaviours**	Perceived value of cleaning	Perceived need and motivation to clean surfaces and hands based on perceived effectiveness	*"I mean, I always washed my hands a lot, obviously, working in a tea room, so I had to wash my hands all the time anyway. So I obviously now wash them for longer, and more carefully. And I also am very, very, very conscious now of whether I'm touching my face or… things like that."* (int 3)
	Perceived value of face-coverings	Perceived need and motivation to wear	*"The thing with the face coverings is, they haven't got any filters in them, these cloth ones…. And I think it*

		face-covering based on perceive effectiveness	*could be more infectious, because it'll get wet with your breathing. And then it's no good to anyone".* (int 6)
	Barrier: Virus is likely to spread before you know you're ill	Belief in the potential to contain the virus if it enters the home	*"do everything in my power to prevent getting it, by assuming that I haven't got it, so wearing a mask in the house, keeping two metres, trying to keep the person that's got it in the household in a separate room"* (int 9)
	Facilitator: Reducing all or nothing thinking	Perception that it is worth reducing exposure to minimise viral load, and that even small changes can make a difference	*"the less we get in touch- the less we have contact with the virus, the safer we will be"* (int 7)
**Acceptability of distancing and isolation**	Barrier: Importance of time together	Includes concerns about own or others' mental well-being if spending time apart, and the value placed on time spent together.	*"I'm worried more about like the mental health of the other people. So although we're very careful, and not mixing. So I don't think I could cut down on the amount of time I spend with other people, because they'll get lonely"* (int 5)<br><br>*"Staying 2m from my 11yr old means me pushing him away and not sharing our only front room seating with him."* (s18)
	Facilitator: Ways of maintaining (distanced) intimacy	Finding ways to maintain emotional intimacy when social distancing or self-isolating	*"We have, I had a bit of, as I said, a bit of a dry cuddle, like I go over his shoulders, but I don't breathe on him and he doesn't breathe on me. So*

For peer review only

			we're kind of on board with it, you know?" (int 2)
**Having capacity to perform protective behaviours**		Factors which reduce a person's capacity to engage with and perform the behaviours. For example; having enough space, or not, in your house to either socially distance or self-isolate. Includes descriptions of encouraging others to adhere to protective behaviours, or the challenges of trying to influence others.	*"My son sits in one settee and my husband and I sit in the other. And that… it doesn't protect us all, we're not all sitting on our own sofa, but who has three sofas in their room? So… we do what we can".* (int 7)<br>*"I will just keep reminding him, all the time, to wash his hands. And he'll say, "I've done it." You say, "No you haven't. The sink's not wet." And, "well I did it. I did do it, I did it when I got to my...' Because he's a sink in his room, "I did it when I got to my room" which we know is not necessarily the case".* (int 1)<br>*"I tried to implement the isolation of amazon parcels and mocked by my teenage daughter!"* (s46)
**Habit forming reduces effort**		Descriptions of behaviours becoming easier when highly practiced/ingrained	*"Anything I can wipe down, I wipe down. So that, now… it, I mean, it is… it still is harder than it used to be, because I never would've done that before. But it is more normal now".* (int 3)<br>*"Good advice needs to be followed but sometimes we forget. It needs to be made into a habit."* (s78)
**Confidence in how to perform the behaviours**		Inconsistent and confusing information undermines confidence. Concise information	*"I've just read an article in the Times that this [washing fresh produce in soapy water] is very dangerous, so I'll have to re-read it and decide, or*

For peer review only

		and validation increases confidence in how to perform the behaviours	*maybe you can tell me, because I've got no idea now, I'm completely confused".* (int 5)<br><br>*"So it's more if that, that's… it's attractiveness of thinking, 'yes, I… you know, if that's the kind of thing I'm doing and, you know, health experts are saying yeah, that's right...*" (int 2)<br><br>*"The advice helps to empower you, that we are not completely defenseless against deadly germs, we can be pro- active in stopping these germs making us ill.."* (s110)
**Social norms affect motivation to engage in the behaviours**		Includes any descriptions about others' perceived behaviour and its' effect on people's willingness to perform effortful behaviours	*"It starts being more relatable, and you can start imagining yourself putting those changes into practice. Like when the guy said his wife is always going round with the anti-bac, you can sort of imagine yourself doing that."* (int 12)

For peer review only

Standards for Reporting Qualitative Research (SRQR)*

http://www.equator-network.org/reporting-guidelines/srqr/

Title and abstract

	Page/line no(s).
**Title** - Concise description of the nature and topic of the study Identifying the study as qualitative or indicating the approach (e.g., ethnography, grounded theory) or data collection methods (e.g., interview, focus group) is recommended	p.1
**Abstract** - Summary of key elements of the study using the abstract format of the intended publication; typically includes background, purpose, methods, results, and conclusions	p.2

Introduction

**Problem formulation** - Description and significance of the problem/phenomenon studied; review of relevant theory and empirical work; problem statement	p.3-4
**Purpose or research question** - Purpose of the study and specific objectives or questions	p.4

Methods

**Qualitative approach and research paradigm** - Qualitative approach (e.g., ethnography, grounded theory, case study, phenomenology, narrative research) and guiding theory if appropriate; identifying the research paradigm (e.g., postpositivist, constructivist/ interpretivist) is also recommended; rationale**	p.9
**Researcher characteristics and reflexivity** - Researchers' characteristics that may influence the research, including personal attributes, qualifications/experience, relationship with participants, assumptions, and/or presuppositions; potential or actual interaction between researchers' characteristics and the research questions, approach, methods, results, and/or transferability	p.7-8
**Context** - Setting/site and salient contextual factors; rationale**	p.5,6,9-8
**Sampling strategy** - How and why research participants, documents, or events were selected; criteria for deciding when no further sampling was necessary (e.g., sampling saturation); rationale**	p.4-6
**Ethical issues pertaining to human subjects** - Documentation of approval by an appropriate ethics review board and participant consent, or explanation for lack thereof; other confidentiality and data security issues	p.8
**Data collection methods** - Types of data collected; details of data collection procedures including (as appropriate) start and stop dates of data collection and analysis, iterative process, triangulation of sources/methods, and modification of procedures in response to evolving study findings; rationale**	p.7-10

For peer review only - http://bmjopen.bmj.com/site/about/guidelines.xhtml

**Data collection instruments and technologies** - Description of instruments (e.g., interview guides, questionnaires) and devices (e.g., audio recorders) used for data collection; if/how the instrument(s) changed over the course of the study	p.7-9
**Units of study** - Number and relevant characteristics of participants, documents, or events included in the study; level of participation (could be reported in results)	p.5,7-9
**Data processing** - Methods for processing data prior to and during analysis, including transcription, data entry, data management and security, verification of data integrity, data coding, and anonymization/de-identification of excerpts	p.8-10
**Data analysis** - Process by which inferences, themes, etc., were identified and developed, including the researchers involved in data analysis; usually references a specific paradigm or approach; rationale**	p.9-10
**Techniques to enhance trustworthiness** - Techniques to enhance trustworthiness and credibility of data analysis (e.g., member checking, audit trail, triangulation); rationale**	p.9.10

Results/findings

**Synthesis and interpretation** - Main findings (e.g., interpretations, inferences, and themes); might include development of a theory or model, or integration with prior research or theory	p.10-22
**Links to empirical data** - Evidence (e.g., quotes, field notes, text excerpts, photographs) to substantiate analytic findings	p.10-22

Discussion

**Integration with prior work, implications, transferability, and contribution(s) to the field** - Short summary of main findings; explanation of how findings and conclusions connect to, support, elaborate on, or challenge conclusions of earlier scholarship; discussion of scope of application/generalizability; identification of unique contribution(s) to scholarship in a discipline or field	p.22-25
**Limitations** - Trustworthiness and limitations of findings	p.24

Other

**Conflicts of interest** - Potential sources of influence or perceived influence on study conduct and conclusions; how these were managed	p.25
**Funding** - Sources of funding and other support; role of funders in data collection, interpretation, and reporting	p.25

*The authors created the SRQR by searching the literature to identify guidelines, reporting standards, and critical appraisal criteria for qualitative research; reviewing the reference lists of retrieved sources; and contacting experts to gain feedback. The SRQR aims to improve the transparency of all aspects of qualitative research by providing clear standards for reporting qualitative research.

For peer review only

**The rationale should briefly discuss the justification for choosing that theory, approach, method, or technique rather than other options available, the assumptions and limitations implicit in those choices, and how those choices influence study conclusions and transferability. As appropriate, the rationale for several items might be discussed together.

Reference:

O'Brien BC, Harris IB, Beckman TJ, Reed DA, Cook DA. **Standards for reporting qualitative research: a synthesis of recommendations.** *Academic Medicine*, Vol. 89, No. 9 / Sept 2014 DOI: 10.1097/ACM.0000000000000388

For peer review only
